# Screening FDA-Approved Oncology Drugs with Three-Dimensional Spheroids Identifies Romidepsin as a Therapeutic Candidate for Osteosarcoma

**DOI:** 10.1158/2767-9764.CRC-25-0121

**Published:** 2025-10-15

**Authors:** Emily E. Seiden, Spencer M. Richardson, Leah A. Everitt, Gabrielle J. Knafler, Alyssa L. Walker, Venetia A. Whiteside, Divya Pillutla, Shrey Ramnath, Gavin P. Kinsella, Piper A. Wilburn, James D. Buschbach, Deep A. Gandhi, M. Reza Saadatzadeh, L. Daniel Wurtz, Patrick J. Getty, Sheldon L. Padgett, Rance M. Gamblin, Michael O. Childress, Christopher M. Fulkerson, Maegan L. Capitano, Karen E. Pollok, Christopher D. Collier, Edward M. Greenfield

**Affiliations:** 1 Department of Orthopaedic Surgery, Indiana University School of Medicine, Indianapolis, Indiana.; 2 Department of Anatomy, Cell Biology, and Physiology, Indiana University School of Medicine, Indianapolis, Indiana.; 3 Indiana University Simon Comprehensive Cancer Center, Indiana University School of Medicine, Indianapolis, Indiana.; 4 Indiana Center for Musculoskeletal Health, Indiana University School of Medicine, Indianapolis, Indiana.; 5 Department of Orthopaedics, University Hospitals Cleveland Medical Center, Case Western Reserve University, Cleveland, Ohio.; 6 Department of Pediatrics, Indiana University School of Medicine, Indianapolis, Indiana.; 7 Herman B. Wells Center for Pediatric Research, Indiana University School of Medicine, Indianapolis, Indiana.; 8 Metropolitan Veterinary Hospital, Akron, Ohio.; 9 Department of Veterinary Clinical Sciences, Purdue University, West Lafayette, Indiana.; 10 College of Veterinary Medicine, Purdue Institute for Cancer Research, Purdue University, West Lafayette, Indiana.; 11 Evan and Sue Ann Werling Comparative Oncology Research Center, Purdue University, West Lafayette, Indiana.; 12 Department of Microbiology and Immunology, Indiana University School of Medicine, Indianapolis, Indiana.; 13 Department of Pharmacology and Toxicology, Indiana University School of Medicine, Indianapolis, Indiana.; 14 Department of Medical and Molecular Genetics, Indiana University School of Medicine, Indianapolis, Indiana.

## Abstract

Osteosarcoma is the most common primary malignant bone tumor and predominantly affects adolescents and young adults. It is the third most common cause of cancer-related deaths among 9 to 24 year olds. Despite aggressive chemotherapeutic and surgical therapies, the survival rate is only 25% for patients with detectable lung metastases at diagnosis and only 70% in patients who present without detectable lung metastases. The poor prognosis is due to growth of metastases irrespective of whether they are initially large enough to detect clinically. It is therefore necessary to develop new methods to target the growth of lung micrometastases. An NCI panel of FDA-approved oncology drugs was therefore screened using three highly metastatic human osteosarcoma cell lines. To more closely approximate *in vivo* micrometastases, the screen used a three-dimensional multicellular *in vitro* osteosarcoma spheroid (sarcosphere) model. Among 13 hits from the initial screen, we identified the histone deacetylase inhibitor (HDI) romidepsin as the most promising inhibitor in secondary screens comparing effects on sarcospheres with clinically achievable levels and to effects on non-transformed cells. Romidepsin potency was evident with and without standard-of-care chemotherapeutics (MAP: methotrexate, adriamycin, and cisplatin) at romidepsin concentrations that are clinically achievable and did not affect non-transformed cells. Romidepsin also substantially outperformed the other three FDA-approved HDIs and eight HDIs in clinical trials. The effects of romidepsin were a transient cell cycle block at G_2_/M and cell death. Importantly, sarcospheres derived from ∼30% of human and 50% of canine patient samples responded to romidepsin at clinically tolerable concentrations (ED_50_s <70 nmol/L).

**Significance:**

Our unbiased sarcosphere-based drug screen identified romidepsin as a promising candidate to repurpose for human and canine patients with metastatic osteosarcoma. This screening strategy allowed us to identify romidepsin-sensitive and -resistant patients. Sarcosphere-based screening may therefore be useful to identify patients most likely to respond clinically to romidepsin or other drugs.

## Introduction

Osteosarcoma is the most common primary malignant bone tumor and is the third most common cause of death in pediatric patients with cancer ages 9 to 24 years (1, 2). With surgery and standard-of-care chemotherapy (consisting of MAP: high-dose methotrexate, doxorubicin, and cisplatin), the 5-year survival rate of patients with osteosarcoma is only 70%, which has not improved since the 1980s (2). For patients with detectable lung metastases, the survival rate is only 25% (1, 3). Lethality in osteosarcoma is due to growth of lung metastases irrespective of whether they are detectable at diagnosis (3, 4). Novel therapeutic agents targeting the growth of both clinically detectable lung metastases and undetectable lung micrometastases are therefore needed to improve outcomes in osteosarcoma. Developing new therapeutics to treat osteosarcoma is challenging for many reasons. Osteosarcoma is genetically complex with substantial heterogeneity between individual patients (5–8). It is therefore unlikely that a single agent will be successful for all patients and personalized treatments may be necessary. Additionally, osteosarcoma is a rare disease, with about 800 new diagnoses per year in the United States, which makes clinical trials difficult and severely reduces commercial interest (9). Repurposing existing FDA-approved drugs is therefore a promising alternative as it reduces the time and cost of drug discovery and provides an opportunity to quickly advance therapies with known safety profiles into clinical trials (10). This study therefore screened the NCI panel of FDA-approved oncology drugs to identify promising agents to repurpose for osteosarcoma.

**Figure 1.**
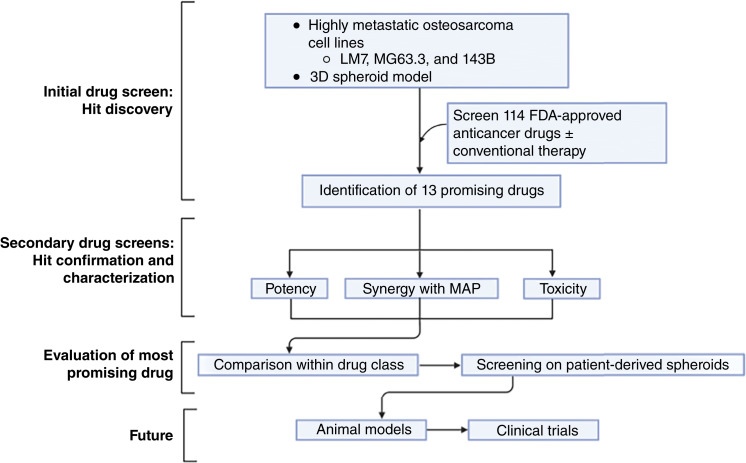
Repurposing promising therapeutics study design. Drug screen used three highly metastatic osteosarcoma cell lines in a 3D sarcosphere platform. The NCI panel of FDA-approved oncology drugs was screened with and without conventional MAP therapy and 13 drugs were identified. The 13 hits were evaluated for potency on sarcospheres with and without MAP therapy and for toxicity on NHOst and SAEC cells. The most promising drug romidepsin was compared with a panel of HDIs that were not included in the initial screen. To further evaluate the top hit, romidepsin was screened on a variety of human and canine patient–derived sarcospheres. Future directions include animal models and canine and human clinical trials. [Created in BioRender. Seiden, E (2025) https://BioRender.com/7tyuopl.]

Three-dimensional (3D) cell culture models more closely represent the *in vivo* tumor environment compared with traditional two-dimensional monolayer cultures and better correlate with *in vivo* responses to chemotherapeutics (11–15). Advantages of 3D models include cell-to-cell interactions and biological gradients of oxygen, nutrients, and metabolic waste products (11, 16–22). 3D models also have genetic profiles and growth kinetics that are more similar to *in vivo* microtumors (11–15). This study therefore used a highly uniform 3D osteosarcoma spheroid (sarcosphere) platform that we previously developed (23) to screen the NCI panel of FDA-approved oncology drugs. Importantly, the platform has acceptable throughput as we routinely obtain hundreds of sarcospheres (one sarcosphere/well) from a single preparation of cells. Screening was performed with and without MAP chemotherapy with sarcospheres derived from three different well-characterized highly metastatic human osteosarcoma cell lines (24). The purpose of this unbiased screen was to identify agents that were potent against sarcospheres at clinically achievable doses, additive to synergistic with MAP chemotherapy, and non-toxic on normal human osteoblasts (NHOst) and nontransformed small airway epithelial cells. Lung cells were included because intranasal therapy is being developed to administer therapeutics directly to lung metastases of patients with osteosarcoma (4, 25, 26). Romidepsin, the most promising drug based on those criteria, was also tested on sarcospheres obtained from low-passage patient-derived cells. Both human and canine patients were included because osteosarcoma is biologically similar in both species (25, 27, 28). For example, canine and human osteosarcomas have similar histology and disease progression and show similar responses to standard therapies (29). In addition, gene expression between human and canine cell lines and pretreated primary tumor samples is similar, indicating that canine osteosarcoma can be used as a model for disease progression (30). In addition, translational studies are facilitated as osteosarcoma is more common in canine patients and canine patients with osteosarcoma have a survival rate of less than 10% (31). The overall layout of our study, including drug screen and hit discovery, hit confirmation and characterization, and evaluation of the most promising drug, is summarized in Fig. 1.

## Materials and Methods

### Cell culture

To best model osteosarcoma metastases, sarcospheres were generated from three cell lines that were chosen because they are the best characterized and most commonly studied human osteosarcoma cell lines that are highly metastatic (24). The 143B cell line (RRID: CVCL_2270; ref. 32), derived from the HOS-TE85 cell line (RRID: CVCL_0439), was obtained from the ATCC. The MG63.3-GFP cell line (RRID: CVCL_WL01; ref. 33), derived from the MG63 cell line (RRID: CVCL_0426), was obtained from the laboratory of C. Khanna DVM, PhD (NCI). The LM7 cell line (RRID: CVCL_0515; ref. 34), derived from the SaOS-2 cell line (RRID: CVCL_0548), was obtained from the laboratory of E.S. Kleinerman, MD (MD Anderson Cancer Center). Small airway epithelial cells (SAEC; ATCC, cat. #PCS-301-010) and NHOst (Lonza, cat. #CC-2538) were used for toxicity screening. All experiments were performed on cells passaged three to seven times after thawing from liquid nitrogen. Cell culture media used were advanced minimum essential medium (Gibco, cat. #12492013) supplemented with 10% FBS (Cytiva, cat. #SH30071.03), 1% GlutaMAX (Gibco, cat. #35050-06), and either 1% penicillin–streptomycin (Gibco, cat. #15140122) or 1% antibiotic–antimycotic (Corning, cat. #30-0004-CL). Medium was sterile filtered before use. All cell cultures were maintained at 37°C in a humidified incubator with 5% CO_2_.

### Isolation of primary cells from patient tumors

Osteosarcoma tissue was obtained during standard-of-care procedures (biopsies or resections) in human patients treated at Indiana University Health and Riley Children’s Hospital with Institutional Review Board approval (#1501467439). Tissue from canine patients with osteosarcoma was obtained from standard-of-care amputations at the Purdue University College of Veterinary Medicine and the Metropolitan Veterinary Hospital. Osteosarcoma diagnosis was confirmed by review of pathology reports. Demographic and clinical information for human and canine patients is in Table 1. Primary osteosarcoma cells were isolated by collagenase/mechanical dispersion using a modification of a previously described protocol (35). Briefly, tissue samples were collected in CO_2_-independent media (Gibco, cat. #18045-088) supplemented with 1% GlutaMax (Gibco, cat. #35050-06) and 1% antibiotic–antimycotic (Corning, Catalog #30-0004-CL) and placed on ice during transportation. The tissue was rinsed with cold PBS (Cytiva, cat. #SH30028.02) and minced prior to incubation in 10 mL of culture media containing collagenase type II (750 units/mL with 13–18 units of clostripain/mL, Worthington, cat. #LS004176) for 3 hours at 37°C and 5% CO_2_. The media and tissue fragments were centrifuged at 200 *g* for 5 minutes. Pellets with a visible layer of red blood cells were then treated with 5 mL of ACK Lysing Buffer (Gibco, cat. #A10492-01) for 1 minute and then centrifuged at 200 *g* for 2 minutes. Pellets were resuspended in culture media, pipetted up and down 15 times to disperse tissue fragments, and centrifuged at 200 *g* for 5 minutes. Finally, any remaining tissue fragments were crushed with a 25-mL pipette and transferred with released cells and culture media to a 100-mm tissue culture dish and incubated at 37°C and 5% CO_2_. Media were changed every 3 days. When a confluence of ∼80% was reached, the cells were passaged to 150-mm dishes at 5,000 cells/cm^2^. Aliquots of the patient-derived osteosarcoma cells were frozen in 10% DMSO (Sigma-Aldrich, D2650), 40% FBS (Cytiva, SH30071.03), and 50% culture media at second passage and stored in liquid nitrogen. The TT2-77 xenoline is a patient cell line that was isolated from a patient-derived xenograft (PDX) that was previously developed and characterized by the Pollok lab (5).

**Table 1.** Clinical information for patients and romidepsin ED_50_s.

Human patients	Sex	Age	Race	Mets at diagnosis	Location	Treatment	% Necrosis	ED_50_ (nmol/L)
Romidepsin sensitive	​	​	​	​	​	​	​	​
IUhOS018B	Male	26	White	No	Humerus	N/A	N/A	1
IUhOS006B	Male	15	Asian	Yes	Femur	N/A	N/A	6
IUhOS1516B	Female	6	White	No	Femur	N/A	N/A	10
TT2-77 Xenoline	Male	20	White	No	Metastasis/pelvis	MAP and IE	90	10
IUhOS020LR	Male	24	Hispanic	Yes	Femur	MAP, IE, Cabo, and GD	40	63
Romidepsin resistant	​	​	​	​	​	​	​	​
IUhOS0410B	Female	9	White	Yes	Femur	N/A	N/A	>1,000
IUhOS0410P	Female	9	White	Yes	Femur	MAP	40	>1,000
IUhOS1516P	Female	6	White	No	Femur	MAP	80	>1,000
IUhOS2226B	Male	16	White	No	Tibia	N/A	N/A	>1,000
IUhOS2226P	Male	16	White	No	Tibia	MAP	90	>1,000
IUhOS1925B	Male	13	Asian	Yes	Tibia	N/A	N/A	>1,000
IUhOS1925P	Male	13	Asian	Yes	Tibia	MAP	40	>1,000
IUhOS002LR	Female	10	White	No	Femur	MAP, IE, and lenvatinib	78	>1,000
IUhOS005B	Female	69	White	No	Femur	N/A	N/A	>1,000
IUhOS013B	Male	15	Hispanic	Yes	Femur	N/A	N/A	>1,000
IUhOS021B	Female	9	White	No	Femur	N/A	N/A	>1,000
IUhOS027P	Female	15	Black	No	Fibula	MAP	10	>1,000
IUhOS029LR	Male	19	Hispanic	No	Thigh	MAP	40	>1,000

Canine patients	Sex	Age	Breed/breed classification	Tumor location	ED_50_ (nmol/L)
Romidepsin sensitive	​	​	​	​	​
MVHcOS004	Male	5 years 2 months	Bernese Mountain Dog/giant	Right distal radius	6
MVHcOS003	Male	6 years 2 months	Great Pyrenees/giant	Right distal tibia	22
PUcOS002	Female	6 years 8 months	Greyhound/large	Right tibia	46
PUcOS004	Male	9 years 4 months	Greyhound/large	Left humerus	57
PUcOS001	Male	8 years 8 months	German Shepherd/large	Distal radius	65
Romidepsin resistant	​	​	​	​	​
MVHcOS001	Female	10 years 4 months	Alaskan Malamute/large	Right distal radius	>1,000
MVHcOS002	Female	7 years 11 months	German Shepherd/large	Right distal radius	>1,000
MVHcOS005	Female	4 years 6 months	Mastiff mix/large	Right distal radius	>1,000
MVHcOS006	Female	5 years 9 months	Cane Corso/giant	Left proximal humerus	>1,000
PUcOS003	Male	11 years 4 months	Mixed breed/unknown	Right proximal femur	>1,000

Anonymized patient codes: 0XX, unmatched samples; B, diagnostic biopsy; c, canine (all from amputations); h, human; IU, Indiana University, Indianapolis, Indiana; LR, local recurrenc; MVH, Metropolitan Veterinary Hospital, Akron, Ohio; OS, osteosarcoma; P, primary resection; PU, Purdue University, West Lafayette, Indiana; XXXX, matched samples from same human patient.

Treatment codes: Cabo, cabozantinib; GD, gemcitabine and docetaxel; IE, ifosfamide and etoposide; N/A, no prior treatment and no % necrosis for biopsy samples.

### 3D *in vitro* sarcosphere generation

Sarcospheres were generated (one sarcosphere/well in a 96-well plate) using the technique we developed previously (23) that was modified from a technique described for epithelial cancers (19). Sarcospheres of the desired size to generate similar gradients of oxygen, nutrients, and metabolic waste to an *in vivo* tumor (400 μm in diameter; ref. 19) were obtained by adjusting the number of cells per well as we previously described (143B: 2,500 cells/sarcosphere, MG63.3-GFP: 1,000 cells/sarcosphere, and LM7: 8,000 cells/sarcosphere; ref. 23). The number of patient-derived cells required to obtain sarcospheres with 400-μm diameters was determined for each patient and varied from 1,000 to 15,000 cells per sarcosphere. For that purpose, sarcosphere sizes were measured using the Incucyte S3 Live Cell Analysis System (Sartorius). Brightfield images were captured using a 4x or 10x objective. Incucyte analysis software was used to automatically detect area of sarcospheres in 96-well plates and diameters were calculated using the formula for the area of a circle. In selected experiments, sarcosphere size was used as an additional measure of drug effects.

Round-bottom 96-well plates were coated with 50 μL/well of 0.5% poly 2-hydroxyethyl methacrylate (Polysciences Inc., cat. #09689-25) in 100% ethanol and incubated at 37°C for 3 days before use to obtain a nonadherent surface. Alternatively, precoated Nunclon Sphera–treated plates (Nunc, cat. #174929) or S-BIO PrimeSurface ultra-low attachment plates (S-BIO, cat. #MS-9096UZ) were used. Highly metastatic osteosarcoma cells cultured in monolayers at 50% to 80% confluence were lifted with Accutase (Innovative Cell Technologies, Inc., cat. #NC9839010), resuspended in media, and diluted to the desired concentration. Cell suspensions were combined with 2.5% Matrigel (Corning, cat. #354234) and 100 μL was added to each well. Three to six wells of the 96-well plate contained 100 μL of 2.5% Matrigel and media without cells were used to provide background values. The peripheral wells of each plate contained PBS or sterile water to minimize evaporation in wells with sarcospheres. Following cell seeding, plates were centrifuged (1,000 *g* for 10 minutes at 4°C) and then incubated at 37°C with 5% CO_2_. Sarcospheres were matured for 24 hours prior to drug treatment (day 0). Then, 100 μL drug treatment or vehicle was added to each well. Plates were incubated for 48 hours and analyzed (day 2). Resazurin reduction on day 2 by vehicle-treated sarcospheres was compared with resazurin reduction on day 0 to calculate sarcosphere growth without drug over 2 days in selected experiments.

### Chemotherapeutics

The panel of 114 FDA-approved oncology drugs (NCI Panel #4804082 and #4803082) was obtained from the NCI Developmental Therapeutic Program at 10 mmol/L in DMSO and stored at −80°C until use. Storage information for the 13 drugs used in the secondary screen is listed in Supplementary Table S1. Romidepsin and all other histone deacetylase (HDAC) inhibitors (HDI; Selleck Chemicals) were dissolved in DMSO and stored at −80°C until use. Cisplatin (Tocris Bioscience) was solubilized in saline to avoid inactivation by DMSO (36). Doxorubicin and methotrexate (Tocris Bioscience) were solubilized in water or PBS, respectively, as done in previous studies from this laboratory (23). Doses of all drugs were obtained following serial dilution from stock solutions. All wells in an experiment received the same concentration of the relevant vehicle.

### Resazurin reduction assays of metabolic activity

Resazurin (Sigma-Aldrich, cat. #R7017-1G) was dissolved in PBS at 0.15 mg/mL. Resazurin solution (20 μL) was added to each well containing sarcospheres in 200 µL of media and to the background wells without sarcospheres. Plates were incubated at 37°C in 5% CO_2_ for 2 or 6 hours. Fluorescence readings were measured at an excitation wavelength of 535 nm and an emission wavelength of 590 nm (Tecan GENios Pro). To calculate metabolic activity, the average fluorescence from the background wells was subtracted from vehicle and treatment wells.

### Initial screen of FDA-approved oncology drugs

Sarcospheres were matured for 24 hours and then treated for 48 hours with media containing 10 µmol/L of each drug and 0.1% DMSO. This relatively high drug concentration was selected to minimize false negatives. Control and background wells also contained 0.1% DMSO. Each drug was tested in duplicate non-adjacent wells to minimize the impact of well position and edge effect. All drugs were tested with and without MAP on sarcospheres derived from the three well-characterized highly metastatic cell lines LM7, 143B, and MG63.3-GFP (24). Effects on metabolic activity are reported as growth inhibition as previously described by the NCI (37) and used by our group (23). Growth inhibition represents a more clinically translatable measure for screening studies in which a value of 0% represents stable disease since the start of treatment, values of 0% to 100% represent progression in which 100% is the untreated control, and values less than 0% represent regression in which −100% represents blank wells without sarcospheres. Drugs from the NCI panel were screened with and without MAP chemotherapeutics. Methotrexate, doxorubicin, and cisplatin were dosed to inhibit sarcosphere growth by 20% by combining the individual chemotherapeutics at a fixed ratio of their individual GIC_50_s (growth inhibition of 50%). This lower concentration of MAP was selected to maintain sufficient dynamic range to identify hits in the screen. Concentrations of MAP for the initial screen are listed in Supplementary Table S2. Screening assay quality was assessed throughout by direct examination of duplicate data which remained highly consistent for all drugs and conditions tested and by calculating Z’ factors, a measure of assay quality in high-throughput screening used to assess assay signal dynamic range and variability (38). The Z’ factor threshold for an “excellent” assay (0.5–1.0) was met for all individual plates and the mean Z’ factor for all plates combined was 0.70 with an SD of 0.10.

### Secondary screens of promising drugs

FDA-approved drugs in the top quartile by the efficacy of our initial screen across all cell lines with and without MAP were considered for inclusion in the secondary screens. An exhaustive literature review was performed for each drug considered. Priority was given to drugs that performed best in the screen across all conditions at clinically achievable concentrations, had unique mechanisms of action, demonstrated efficacy in other solid cancers, had favorable or untested efficacy in osteosarcoma basic research or clinical trials, and were not already recommended as first- or second-line therapies for patients with osteosarcoma by the National Comprehensive Cancer Network. This process identified 13 promising drugs for the secondary screens evaluating potency, toxicity, and potential for combination therapy with MAP. For these secondary analyses, we reported drug responses as the percent of control in which 0% represent complete inhibition and 100% represent no response compared with the untreated control. This analysis allowed us to more accurately pool results from three to six independent experiments as values do not fluctuate based on metabolic activity at the start of treatment. Potency experiments were performed on sarcospheres following serial dilution of each drug at log-based concentrations from 0.001 to 10 μmol/L to determine the ED_50_ (effective dose at which metabolic activity is 50% of the control). Combination therapy experiments were performed by comparing drug response across multiple doses with and without MAP at a fixed dose. MAP was dosed to obtain 50% reduction in metabolic activity by combining each individual chemotherapeutic at a ratio of their individual ED_50_s. Concentrations of MAP used in secondary screen are listed in Supplementary Table S2. Toxicity experiments were performed on monolayers of non-transformed NHOst and SAEC cells at 50% to 80% confluency in similar fashion to calculate the TD_50_ (toxic dose at which metabolic activity is 50% of the control).

### Cell cycle analysis

Thirty sarcospheres treated with 10 nmol/L romidepsin or vehicle were collected and pooled after 24 hours of treatment. Sarcospheres were washed with PBS to remove any media and then resuspended in Accumax Cell Detachment Solution (STEMCELL Technologies, #07921) for 15 to 30 minutes. Sarcospheres were manually dissociated by pipetting up and down at least 10 times. Following dissociation, cells were fixed in Cytofix/Cytoperm (BD Cytofix/Cytoperm Fixation/Permeabilization Solution Kit, BDB554714) for 10 to 20 minutes at 4°C protected from light. Fixation solution was removed with two washes in sterile PBS. Cells were resuspended in PBS and stored for no more than 72 hours at 4°C. Cells were transferred to flow cytometry tubes for staining. For DAPI staining, 1 mg/mL DAPI (BD Pharmingen, #564907) was diluted 1000-fold in sterile PBS and 500 μL was added to the appropriate tubes containing an equal volume of cell suspension. Flow cytometry was performed using a BD LSRFortessa X-20 flow cytometer using a 407 laser. Cell cycle analysis was performed using the Watson model in FlowJo software (version 10.10.0).

### Transient romidepsin treatment

Sarcospheres were generated and treated with various doses of romidepsin and resazurin reduction assays of metabolic activity were conducted as described above on days 2, 4, 8, and 14 after romidepsin addition. Romidepsin was removed after 24 hours with three cycles of media exchange using a MultiFlo FX Multimode Dispenser with Automated Media Exchange module (BioTek, Agilent). As each cycle exchanged 87.5% of the media, three cycles would remove 99.8% of the romidepsin. Sarcosphere losses were minimized by removing the media stepwise (25 μL/step, 10 μL/second) and by centering the sarcospheres in the middle of each well by centrifugation (1,000 *g* for 6 minutes) before each media exchange cycle. After each assay of metabolic activity, resazurin was removed by three cycles of media exchange and the media were changed on days 10 and 12 by one cycle.

### Statistical analysis

For the initial screen of FDA-approved drugs, unsupervised hierarchical clustering of cell lines based on drug responses was used to generate a heatmap and a sample dendrogram. Based on the mean response, the drugs were sorted. The R-project (version 3.1.2.) hclust function with the Ward-D2 method was used to generate the dendrogram. Dendrogram at bottom illustrates the arrangement of the cell lines based on comparison of responses via hierarchical clustering. The height of the tree branch connecting end nodes (cell lines) in the dendrogram is proportional to the value of the intergroup dissimilarity. The color bar on the left of the heatmap shows the classification of the drugs.

All other data are presented as a mean of at least three independent experiments. Error bars represent SDs. Each experiment included a minimum of three sarcospheres per group. ED_50_s and TD_50_s were calculated using a three-parameter logistic regression analysis (GraphPad Prism version 10.2.3). Heat maps were ranked by the means of each row in the heat map (highest to lowest value). Blue indicates the highest value of a heatmap, red is the lowest, and white indicates the median of the row means. Synergy scores for Bliss and Loewe models of additivity were calculated using SynergyFinder+ (39). One-way ANOVA with a Dunnett multiple comparisons test was used to compare treated groups with control in dose–response experiments and to compare groups for cell cycle analysis (GraphPad Prism version 10.2.3). Two-way ANOVA with a Šídák multiple comparisons test (GraphPad Prism version 10.2.3) was used to compare treated groups with control groups in preliminary screen of the other HDIs (FDA approved and in clinical trials). A Friedman one-way repeated measures ANOVA by ranks (Friedman test) with a Dunn multiple comparisons test was used to compare each group over time for sarcosphere time course experiments and romidepsin recovery experiments (GraphPad Prism version 10.2.3). All graphs and heatmaps were generated using GraphPad Prism (version 10.2.3).

## Results

### HDI, proteasome inhibitors, and topoisomerase inhibitors were most effective in initial screen

**Figure 2.**
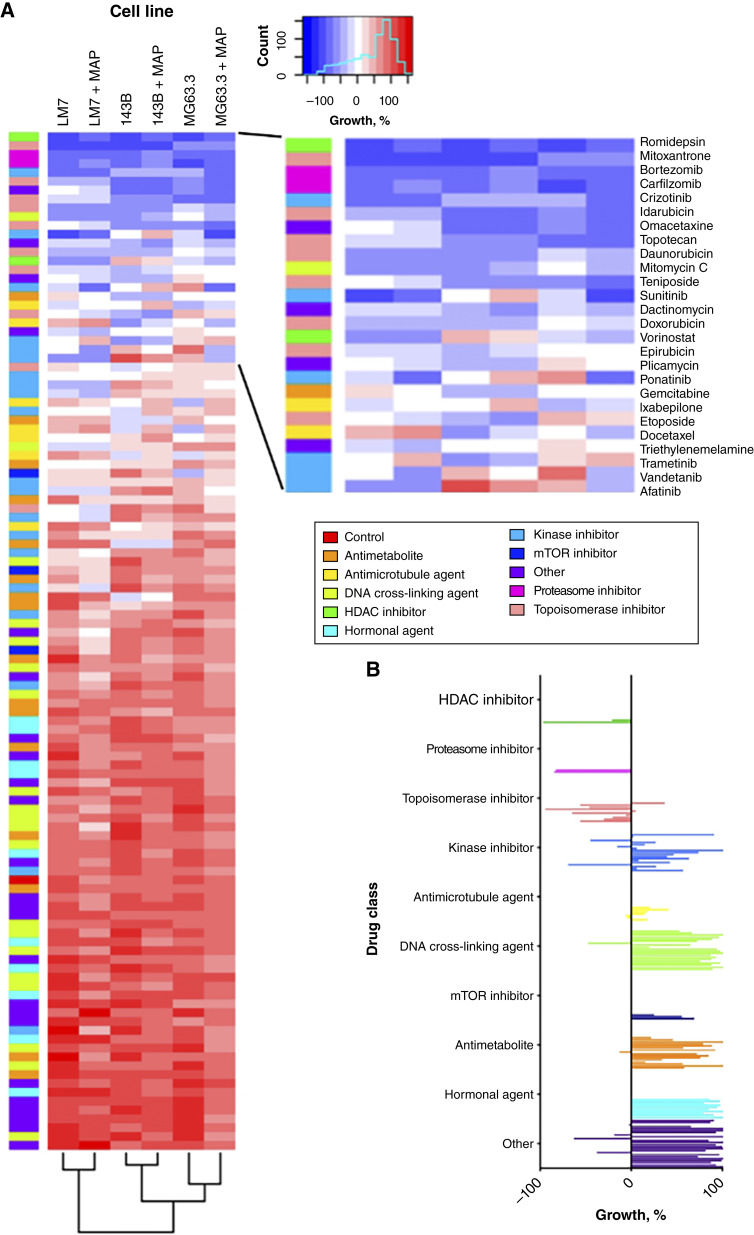
Top hits of initial screen are HDIs, proteasome inhibitors, and topoisomerase inhibitors. **A,** Screen of FDA-approved oncology drugs on sarcospheres generated from 143B, MG63.3-GFP, and LM7 cell lines. Heatmap shows inhibition of growth in blue and little or no effect in red. Colored bars on left of figure indicate control or drug class. Results of unsupervised clustering analysis are shown at the bottom of the figure. Sarcospheres were screened with the individual drugs (10 μmol/L) both with and without MAP chemotherapeutics for 48 hours. All wells contained 0.1% DMSO as vehicle. Growth was assessed by change in metabolic activity during the 48-hour treatment period. 0% represents stable disease since the start of treatment, 0% to 100% represents progression in which 100% is the untreated control, and values less than 0% represent regression for which −100% represents blank wells without sarcospheres. **B,** Summary of the initial screen showing the mean of the rows for each drug in **A**.

In the initial unbiased screen, the NCI panel of FDA-approved oncology drugs was tested at 10 μmol/L against sarcospheres derived from three highly metastatic human osteosarcoma cell lines (143B, MG63.3-GFP, and LM7). Dark blue in heatmap indicates inhibition of sarcosphere growth based on resazurin reduction assays of metabolic activity (Fig. 2A). Unsupervised clustering analysis showed that 143B and MG63.3-GFP sarcospheres responded relatively similarly, whereas responses by LM7 sarcospheres were relatively distinct (Fig. 2A). HDIs, proteasome inhibitors, and topoisomerase inhibitors were most effective both with and without MAP chemotherapeutics in sarcospheres generated from all three osteosarcoma cell lines (Fig. 2A and B). Few drugs from the other drug classes reduced metabolic activity (Fig. 2B). In agreement with our previous study of the osteosarcoma standard-of-care chemotherapeutics (23), doxorubicin was effective with sarcospheres derived from all three cell lines and methotrexate was effective with 143B sarcospheres. In contrast, cisplatin had no detectable effect on sarcospheres derived from any of the cell lines, presumably because it was inactivated during preparation of the NCI panel by solubilization in DMSO (36).

### Secondary screens identified the HDI romidepsin as the most promising drug to repurpose for osteosarcoma

**Figure 3.**
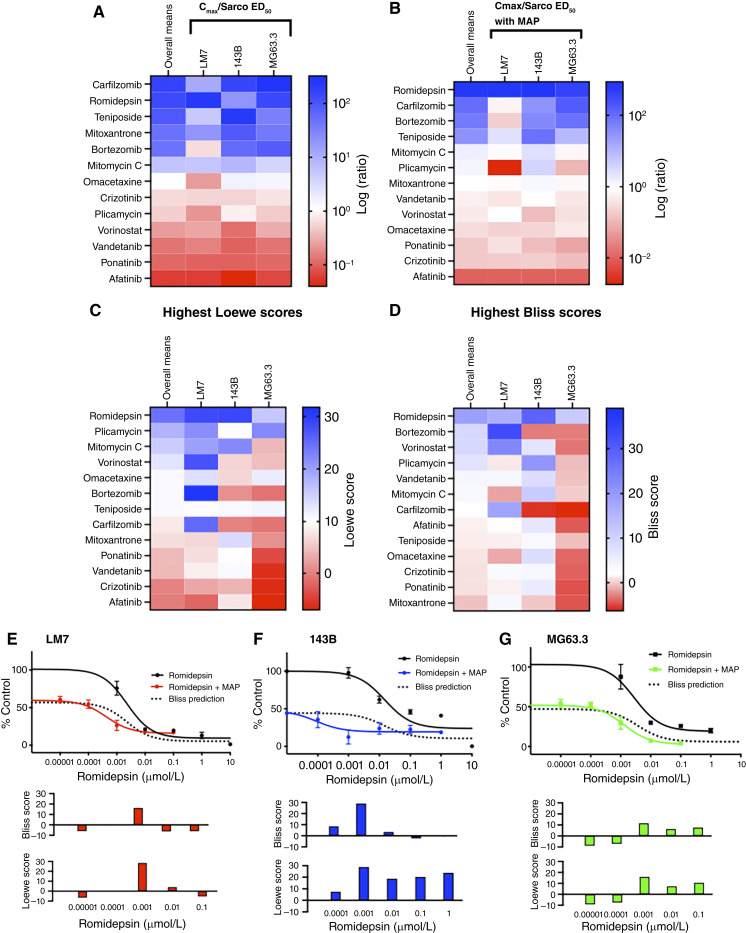
The HDI romidepsin is most promising of the 13 FDA-approved oncology drugs in the secondary screen. All heatmaps in this figure are sorted by the overall row means shown in the first column of each heatmap. Dark blue indicates highest values (most favorable), dark red the lowest values (least favorable), and white is set at the median of the row means. **A** and **B,** Ratios of Cmax/sarcosphere (Sarco) ED_50_ with and without MAP reflect potency at clinically achievable concentrations. Cmax and ED_50_s are shown in Supplementary Table S3 and Supplementary Fig. S1. ED_50_s were determined for sarcospheres derived from LM7, 143B, and MG63.3-GFP cell lines in *N* = 3 independent dose–response experiments with each drug. Each experiment included at least three sarcospheres per group. **C** and **D,** Synergy scores in combination with MAP. Highest synergy scores at concentrations less than Cmax were calculated for each drug using the Bliss and Loewe models of additivity from the data shown in **E–G** and Supplementary Figs. S2–S4. **E–G,** Romidepsin dose responses with and without MAP for sarcospheres derived from LM7, 143B, and MG63.3-GFP cell lines. Solid black lines indicate romidepsin dose responses without MAP, and colored lines indicate romidepsin dose response with MAP. Dotted lines indicate Bliss prediction of perfect additivity. Bar graphs show synergy scores at each individual drug concentration.

After exclusion of the standard-of-care doxorubicin and its analogues (idarubicin, daunorubicin, and epirubicin), 13 of the remaining 22 most effective drugs in the initial screen were selected for secondary screening. Priority was given to drugs that performed best in the screen across all conditions at clinically achievable concentrations, had unique mechanisms of action, demonstrated efficacy in other solid cancers, had favorable or untested efficacy in osteosarcoma basic research or clinical trials, and were not already recommended as first- or second-line therapies for patients with osteosarcoma by the National Comprehensive Cancer Network. The 13 drugs included both HDIs (romidepsin and vorinostat) and both proteasome inhibitors (bortezomib and carfilzomib) in the initial screen, as well as inhibitors of tyrosine kinases (afatinib, crizotinib, ponatinib, and vandetanib), topoisomerase (mitoxantrone and teniposide), transcription (plicamycin) and translation (omacetaxine), and a DNA cross-linker (mitomycin C). Dose–response experiments confirmed the initial screening results for all 13 drugs (Supplementary Fig. S1A). Bortezomib had the lowest ED_50_ with 143B and MG63.3-GFP sarcospheres, whereas romidepsin had the lowest ED_50_ with LM7 sarcospheres and the second and third lowest with MG63.3-GFP and 143B sarcospheres, respectively (Supplementary Fig. S1B–S1D; Supplementary Table S3). The ED_50_s were also compared with the clinically achievable level (Cmax) of each drug, listed in Supplementary Table S3 (40–44), which represents the peak plasma concentration measured and tolerated clinically. Carfilzomib had the highest overall mean of the three Cmax/ED_50_ ratios and romidepsin ranked second (Fig. 3A). A comparison of the Cmax/ED_50_ ratios of sarcospheres from each cell line showed that romidepsin had the highest ratio with LM7 sarcospheres, the second highest ratio with MG63.3-GFP sarcospheres, and the fifth highest with 143B sarcospheres (Fig. 3A).

The 13 drugs were also screened in combination with the three MAP chemotherapeutic drugs because combination therapy with MAP is a likely application for any new osteosarcoma drug. When ED_50_s were measured in the presence of MAP, romidepsin had the most favorable Cmax/ED_50_ ratio in sarcospheres derived from all three cell lines (Fig. 3B). Moreover, by the Bliss and Loewe additivity models (45), romidepsin had the highest overall mean of synergy scores of the 13 drugs at concentrations less than Cmax (Fig. 3C and D). In addition, romidepsin had additive-to-synergistic activity in combination with MAP in sarcospheres derived from all three cell lines. Additive-to-synergistic activity is indicated in Fig. 3E–G by downward shifts in the romidepsin dose responses in combination with MAP compared with the Bliss additivity model depicted by the dotted lines. Importantly, the highest synergy scores for romidepsin were at 1 nmol/L, which is substantially less than its Cmax of ∼700 nmol/L (Fig. 3E–G bar graphs). In comparison, the only other examples of additive-to-synergistic activity in combination with MAP by Bliss curve fitting were ponatinib and vorinostat with LM7 sarcospheres and plicamycin with 143B sarcospheres (Supplementary Figs. S2–S4).

**Figure 4.**
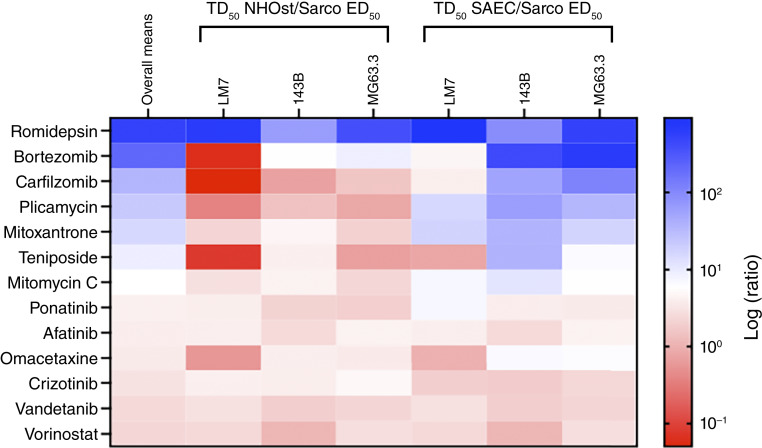
Romidepsin has the largest overall therapeutic index of all 13 hits from the initial screen. Data represent dose–response experiments for top 13 hits from initial screen. Ratios of TD_50_/sarcosphere (Sarco) ED_50_ reflect therapeutic indices with NHOst and non-transformed human SAEC. White on the color scale is set at the median of the overall row means, shown in the first column. TD_50_s, doses that reduce metabolic activity of NHOst and SAEC by 50%, are shown in Supplementary Figs. S5A and S6A. The TD_50_/ED_50_ ratios are shown in Supplementary Figs. S5B–S5D and S6B–S6D. TD_50_s were determined in *N* = 3 independent dose–response experiments with each drug. Each experiment included at least three culture wells per group. ED_50_s, doses that reduce metabolic activity of 143B, LM7, or MG63.3-GFP sarcospheres by 50%, are shown in Supplementary Fig. S1B–S1D and Supplementary Table S3.

To evaluate the safety of the 13 drugs, we measured effects on metabolic activity of monolayers of NHOst and non-transformed human SAEC (Fig. 4). We elected to use monolayers for this purpose rather than 3D spheroids because neither osteoblasts nor SAECs form 3D aggregates *in vivo*. SAEC were included because aerosol therapy is being developed to directly treat lung metastases of patients with osteosarcoma (26, 46, 47). Six therapeutic indices were calculated for each drug as the TD_50_ for NHOst and SAEC divided separately by each of the three sarcosphere ED_50_s. Romidepsin had the largest overall mean of the therapeutic indices on NHOst and SAEC cells (Fig. 4). Romidepsin had 11-, 42-, and 162-fold higher NHOst therapeutic indices than the next highest drug in 143B, MG63.3-GFP, and LM7 sarcospheres, respectively (Fig. 4; Supplementary Fig. S5A–S5D). Romidepsin also had the largest individual SAEC therapeutic index with LM7 sarcospheres and the second largest with 143B and MG63.3-GFP sarcospheres (Fig. 4; Supplementary Fig. S6A–S6D). Taken together, the potency and toxicity screens identified the HDI romidepsin as the most promising drug to repurpose for osteosarcoma. The next most promising FDA-approved drugs were bortezomib and carfilzomib, the only two proteasome inhibitors in the NCI panel.

### Romidepsin is more promising than all other tested HDIs

**Figure 5.**
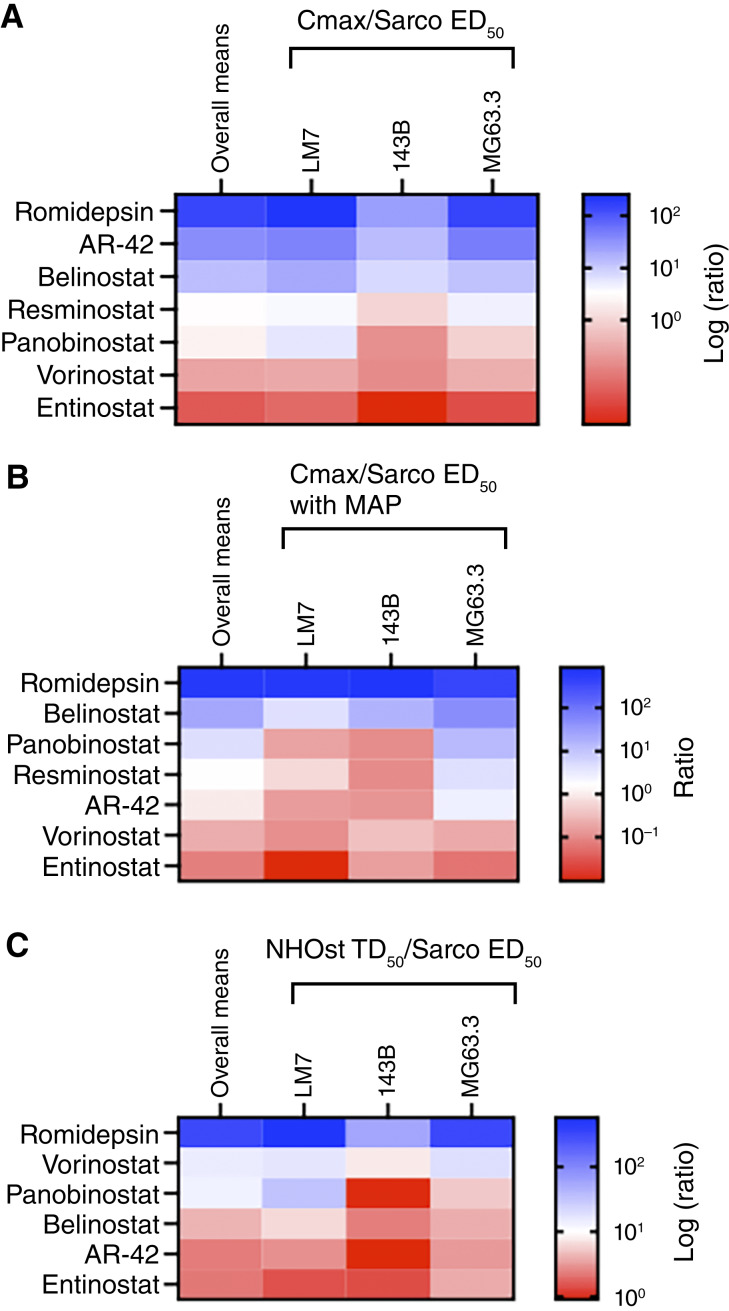
Romidepsin is more promising than all other tested HDIs. Romidepsin was compared with the three other FDA-approved HDIs, as well as eight that are in clinical trials. Five of the HDIs in clinical trials (abexinostat, givinostat, mocetinostat, pracinostat, and quisinostat) weakly inhibited sarcosphere metabolic activity when tested at their Cmax in preliminary studies and were therefore excluded from further study (Supplementary Fig. S7). All heatmaps in this figure are sorted by the overall row means shown in the first column of each heat map. Dark blue indicates highest values (most favorable), dark red the lowest values (least favorable), and white is set at the median of the row means. **A** and **B,** Ratios of Cmax/sarcosphere (Sarco) ED_50_ with and without MAP reflect potency at clinically achievable concentrations. Cmax and dose responses are shown in Supplementary Table S3 and Supplementary Fig. S8. ED_50_s were determined for sarcospheres derived from LM7, 143B, and MG63.3-GFP cell lines in *N* = 3 independent dose–response experiments with each drug. Each experiment included three sarcospheres per group. **C,** Ratios of TD_50_/sarcosphere (Sarco) ED_50_ reflect therapeutic indices with NHOst. TD_50_s were determined in *N* = 3 independent dose–response experiments with each drug. Each experiment included at least three culture wells per group.

Each HDI has unique pharmacokinetics and pharmacodynamics, targets different classes of HDACs, and therefore could be more effective or safer than romidepsin (48–54). As only two HDIs were included in the initial screen, romidepsin was compared with the three other HDIs that are FDA approved for oncology patients and seven that are in clinical trials. The HDIs in clinical trials were initially screened at their Cmax, with and without MAP chemotherapeutics, against sarcospheres derived from each of the osteosarcoma cell lines. AR-42 and resminostat were most effective in those preliminary studies (Supplementary Fig. S7) and were therefore advanced for further study with the FDA-approved HDIs (belinostat, panobinostat, romidepsin, and vorinostat). Dose–response experiments with sarcospheres derived from all three cell lines showed that romidepsin had the lowest ED_50_s with and without MAP chemotherapeutics (Supplementary Table S3 and Supplementary Fig. S8), the most favorable Cmax/ED_50_ ratios with and without MAP (Fig. 5A and B), and the largest NHOst therapeutic indices (Fig. 5C). Moreover, the effects of romidepsin and vorinostat in this screen are very similar to, and therefore confirm, the effects of those HDIs in the secondary screen (Supplementary Table S3). Therapeutic indices for the HDIs were not determined with the SAECs because they were less informative than the NHOst therapeutic indices for the original 13 drugs (Fig. 4).

### Primary responses to romidepsin by cell line–derived sarcospheres are dependent on cell line

**Figure 6.**
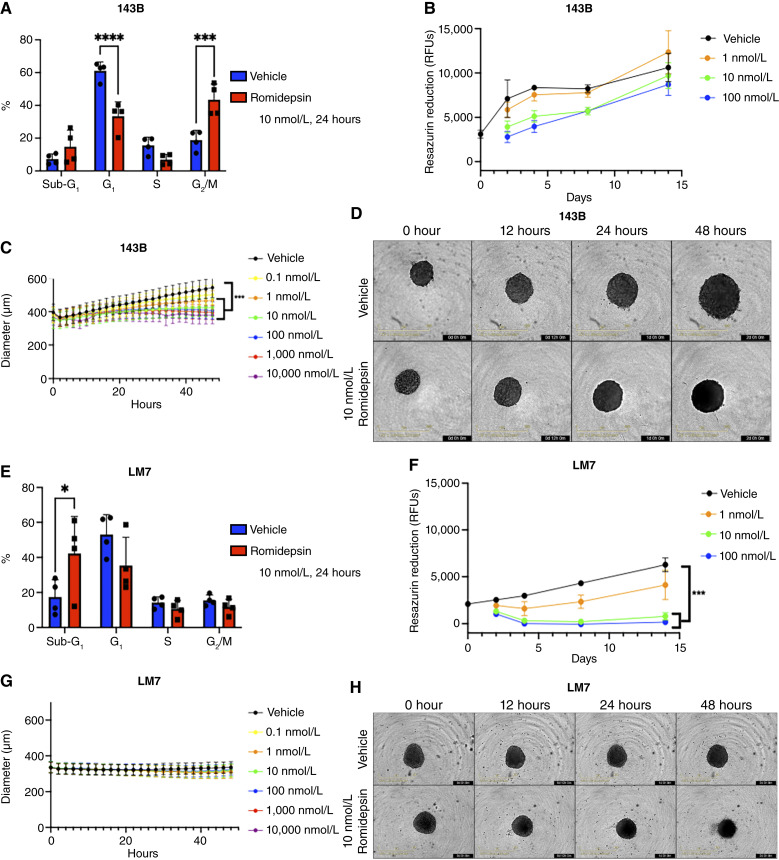
The primary effects of romidepsin are a transient block in proliferation for 143B sarcospheres and cytotoxicity for LM7 sarcospheres. 143B (**A–D**) and LM7 (**E–H**) **s**arcospheres were exposed to the indicated concentrations of romidepsin or vehicle for 24 hours (**A**, **B**, **E**, and **F**) or the indicated times (**C**, **D**, **G**, and **H**). **A** and **E,** Cell cycles were analyzed by flow cytometry after exposure to 10 nmol/L romidepsin or vehicle for 24 hours. Each symbol represents the percentage of the cell population in each phase from an individual experiment with 30 sarcospheres per group. Colored bars show means ± SD of *N* = 3–4 independent experiments. A one-way ANOVA with a Dunnett multiple comparisons test was used to compare experimental groups with the vehicle control group *, *P* < 0.05; ***, *P* < 0.01; ****, *P* < 0.001. **B** and **F,** Recovery from a 24-hour romidepsin exposure was assessed by repeated measurements of metabolic activity on days 2, 4, 8, and 14 after romidepsin addition. All repeated measurements were on the same set of sarcospheres, except that measurements on day 0 were on a separate plate of sarcospheres. Each symbol represents mean ± SD of *N* = 3 independent experiments. Each experiment included six sarcospheres per dose group. Friedman one-way repeated measures ANOVA by ranks with a Dunn multiple comparisons test was used to compare each experimental group with the vehicle control group over time. ***, *P* < 0.001. **C** and **G,** Sarcosphere diameters were measured every 2 hours during exposure to the indicated concentrations of romidepsin or vehicle by the Incucyte S3 Live Cell Analysis System (Sartorius). Each symbol represents mean ± SEM of *N* = 3–4 independent experiments. Each experiment included 3–6 sarcospheres per dose group. A Friedman test with a Dunn multiple comparisons test was used to compare each experimental group with the vehicle control group for 143B sarcosphere size experiments: *, *P* < 0.05 (1 nmol/L group), ***, *P* < 0.001 (10–10,000 nmol/L groups). **D** and **H,** Representative images from experiments depicted in **C** and **G**. Yellow size bars on the lower left of images show 800 mm. Timestamp is indicated in the lower right of each image.

Interestingly, the primary response to romidepsin differs between 143B and LM7 sarcospheres. Exposure to 10 nmol/L romidepsin for 24 hours induced a G_2_–M cell cycle block in 143B sarcospheres without altering the proportion of sub-G_1_ cells (Fig. 6A). Consistent with a cell cycle block, 143B sarcospheres rapidly recovered from a 24-hour exposure to romidepsin so that there was little or no effect on metabolic activity 1 to 2 weeks later (Fig. 6B). Romidepsin also inhibited the increase in size of the 143B sarcospheres that occurs during the initial 48-hour observation period (Fig. 6C and D). In contrast to 143B sarcospheres, exposure to 10 nmol/L romidepsin increased the proportion of sub-G_1_ cells in LM7 sarcospheres without inducing a cell cycle block (Fig. 6E). Consistent with increased cell death, exposure of LM7 sarcospheres for 24 hours to clinically tolerable doses of romidepsin (10–100 nmol/L) had a sustained effect for 14 days on metabolic activity without evidence of recovery (green and blue lines in Fig. 6F). The size of vehicle-treated LM7 sarcospheres did not detectably change during the initial 48-hour period (black line in Fig. 6G and H). Those results are consistent with the similar levels of metabolic activity before and after 48 hours in the vehicle-treated group (black symbols in Fig. 6F). Interestingly, romidepsin did not alter size of LM7 sarcospheres during the same 48-hour period (Fig. 6G and H) despite a potent decrease in metabolic activity (Figs. 3E and 6F; Supplementary Fig. S1; Supplementary Table S3). Taken together, the results in this paragraph suggest that the primary effects of romidepsin are a transient block in proliferation for 143B sarcospheres and cytotoxicity for LM7 sarcospheres.

### Sarcospheres derived from a subset of human and canine patients with osteosarcoma are sensitive to romidepsin

**Figure 7.**
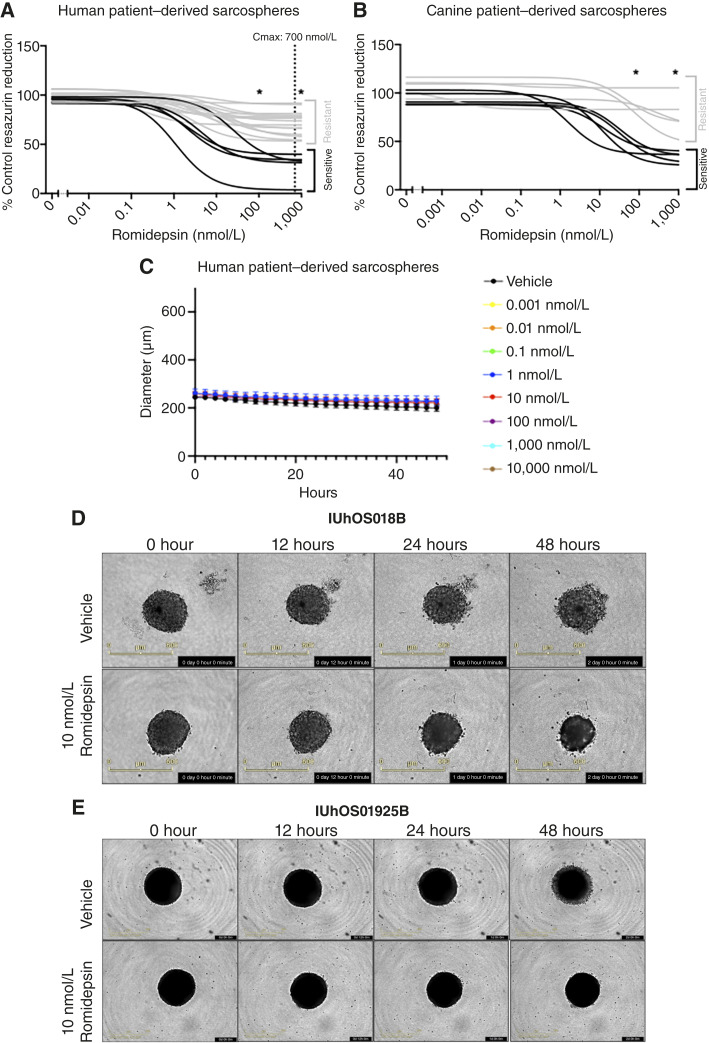
Sarcospheres from a subset of patients are sensitive to romidepsin. Metabolic activity of sarcospheres from 18 human (**A**) and 10 canine (**B**) patients was measured after exposure for 48 hours to the indicated concentrations of romidepsin. Black curves represent sensitive subset of patients with ED_50_s <70 nmol/L. Gray curves represent resistant subset, defined as ED_50_ >1,000 nmol/L. Each curve represents the mean of at least *n* = 3 independent experiments with sarcospheres from an individual patient sample. Each experiment included six sarcospheres at each of the indicated doses. Asterisks (*) indicate dose groups (100 and 1,000 nmol/L) that are significantly different (*P* < 0.05) from the vehicle-treated group for all sensitive samples based on one-way ANOVA with Dunnett multiple comparisons tests. **C,** Diameters of human patient–derived sarcospheres were measured every 2 hours during exposure to the indicated concentrations of romidepsin or vehicle by the Incucyte S3 Live Cell Analysis System (Sartorius). Each symbol represents mean ± SEM of *N* = 6 patients (two romidepsin sensitive and four romidepsin resistant). Sarcospheres from each patient were analyzed in 1–3 independent experiments. Each experiment included 3–6 sarcospheres per dose group. **D,** Representative images of romidepsin-sensitive sarcospheres from experiments depicted in **C**. Yellow size bars in lower left of images show 600 mm. Time stamp is shown in lower right of each image. **E,** Representative images from romidepsin-resistant patients from experiments depicted in **C**. Yellow size bars in lower left of images show 800 mm. Time stamp is shown in lower right of each image.

Clinical information for the human and canine patients with osteosarcoma is shown in Table 1. Human samples included primary biopsies, resections, local recurrences, and metastatic sites, which were representative of what is seen in the clinic. All canine samples were from standard-of-care amputations. To determine whether the isolated cells are primarily derived from the osteosarcoma itself, rather than from bystander cells, we analyzed copy-number variations by whole-exome sequencing. As expected for osteosarcoma, numerous amplifications and deletions were found in cells isolated from the analyzed human patients (Supplementary Fig. S9). Effects of romidepsin on metabolic activity were measured in sarcospheres generated from low-passage cells isolated from the human and canine osteosarcoma samples. Sarcospheres from five of 18 human samples and five of 10 canine samples responded to romidepsin with ED_50_s of 1 to 65 nmol/L, which is 10- to 700-fold less than the Cmax in humans of ∼700 nmol/L (black curves in Fig. 7A and B; Table 1; ref. 40). These groups were considered sensitive to romidepsin. All but one of the sensitive patients had ED_50_ <20 nmol/L and one patient was notably more sensitive (ED_50_ = 1 nmol/L). In contrast, sarcospheres from the other patient samples had ED_50_s greater than 1,000 nmol/L and were considered resistant to romidepsin (gray curves in Fig. 7A and B and Table 1). Romidepsin sensitivity was similar among human samples obtained from primary biopsies and posttreatment samples (primary resections, local recurrences, and metastases) as three of nine pretreatment samples were sensitive and two of nine posttreatment samples were sensitive (Table 1). Overall, a subset of human and canine patient–derived sarcospheres was found to be sensitive to romidepsin. Those results suggest that sarcosphere-based screening may be useful to identify patients most likely to respond clinically to romidepsin. Sizes of both romidepsin-sensitive and romidepsin-resistant sarcospheres were measured during exposure to vehicle or various doses of romidepsin. Similarly to the results of the LM7 sarcospheres (Fig. 6G and H), the vehicle-treated and the romidepsin-treated patient-derived sarcospheres did not change in size during the 48-hour observation period (Fig. 7C–E; Supplementary Fig. S10) despite the potent decrease in metabolic activity in response to romidepsin (black curves in Fig. 7A and B and Table 1).

## Discussion

Long-term survival in osteosarcoma has not improved over the past four decades because of therapy-resistant lung metastases, indicating a need for new therapies targeting metastatic disease. We previously developed a high-throughput sarcosphere screening platform to identify promising therapeutics for metastatic osteosarcoma (23). The current study measured effects of FDA-approved oncology drugs on the sarcospheres with or without MAP chemotherapeutics. The resultant ED_50_ values were compared with Cmax values from the literature and with toxicity measured on NHOst and non-transformed human airway epithelial cells. Romidepsin, an HDI that is FDA approved for the treatment of cutaneous T-cell lymphoma (55, 56), was the most promising candidate to repurpose for osteosarcoma among the NCI panel of 114 FDA-approved oncology drugs. The next most promising FDA-approved drugs were bortezomib and carfilzomib, the only two proteasome inhibitors in the NCI panel. Romidepsin also substantially outperformed the three other FDA-approved HDIs and eight HDIs that are in clinical trials. Romidepsin also potently inhibited metabolic activity of sarcospheres derived at low passage from ∼30% of human and 50% of canine patient–derived samples with ED_50_s substantially less than the Cmax in human patients. Our finding that sarcospheres from individual patients differ in romidepsin sensitivity suggests that sarcospheres might be useful as avatars to identify patients with osteosarcoma most likely to respond clinically to romidepsin. In support of that possibility, 3D spheroid responses can predict clinical drug responses in multiple other cancers (57–60). Although our screen incorporated a broad array of anticancer agents, drugs that were FDA approved more recently or are approved only for non-oncological conditions, as well as antibody-based drugs and investigational drugs other than HDIs, were not included. Future studies should build upon our findings by evaluating additional compounds.

The results of our unbiased, sarcosphere-based, drug screen are broadly consistent with previous osteosarcoma drug screens. For example, romidepsin, panobinostat, bortezomib, and carfilzomib were among the top hits in four independent screens with monolayers of either established osteosarcoma cell lines or PDX cell lines (61–64). Moreover, one of the cell line screens also included drug combinations, and five of the top six combinations were romidepsin with either bortezomib, carfilzomib, or the Wee1 kinase inhibitor MK1775, as well as panobinostat with carfilzomib (62). MK1775 was not included in our screen as it is not approved by the FDA. An advantage of one of the cell line monolayer screens was that dosages and exposure times were based on Cmax and half-lives of each drug in patients (62). However, a limitation of monolayer screens is the inability to capture the complexity of an *in vivo* tumor because they do not model cell-to-cell interactions or biological gradients (11, 16–22). We therefore view sarcospheres as a more clinically relevant model of micrometastatic lung metastases worth the somewhat lower throughput when compared with traditional monolayer models for drug screening.

Other investigators have also used 3D models to overcome the limitations of monolayers for osteosarcoma drug screens. For example, 28 patients with osteosarcoma were included in a screen based on organoids of patient-derived cells in Matrigel (65). Although romidepsin was not included in that screen, five drugs were more effective at 1 μmol/L against osteosarcomas than other sarcomas. Of those drugs, topotecan ranked eighth in our initial screen at 10 μmol/L but was not advanced to our secondary screen as its Cmax is only 6.5 nmol/L (66). Cabozantinib ranked fifteenth against LM7 sarcospheres in our initial screen but had little or no effect on 143B or MG63.3 sarcospheres and everolimus had little or no effect on sarcospheres derived from all three of the cell lines. Moreover, topotecan, cabozantinib, and everolimus are already included in the National Comprehensive Cancer Network list of “Other Recommended Regimens” for osteosarcoma second-line therapy. Finally, cediranib and ceralasertib were not included in our screen as they are not approved by the FDA. An additional limitation of that study is that the number of drugs that could be screened was restricted by the number of cells obtained from each patient and therefore ranged from one to 26 drugs per osteosarcoma sample (65). In a screen of spheroids derived from 65 pediatric patients with various types of cancer, including seven osteosarcomas, panobinostat and bortezomib ranked fifteenth and nineteenth, respectively, of 75 drugs (67). However, that screen did not include romidepsin or carfilzomib and the osteosarcoma results were not reported separately from the other types of cancer. Moreover, drug addition preceded spheroid formation unlike our screen in which drugs were added to pre-formed sarcospheres, which is more translationally relevant as most patients with osteosarcoma already have lung metastases at the time of diagnosis (3, 4). In addition to the large drug screens described in this paragraph, a small number of FDA-approved oncology drugs were tested against a small number of osteosarcoma PDXs (bioRxiv 2024.2007.2022.604648; refs. 5, 6, 68–70). However, none of those studies evaluated romidepsin, bortezomib, or carfilzomib.

In cancers with targetable driver mutations, genomic approaches can be used to select drugs for targeted therapy. However, this approach risks both false-positive and false-negative drug predictions in a disease without driver mutations and as genetically complex as osteosarcoma (5–8). With further validation, emerging omics-based approaches might nonetheless be combined with patient-derived sarcospheres to identify personalized therapeutics for patients with osteosarcoma. Genomics have already successfully identified pathways for targeted therapy of patient-derived osteosarcoma xenografts (5, 6, 68, 70). Although PDXs provide a useful model to study drug effects *in vivo*, multiple roadblocks need to be overcome before they can successfully inform personalized approaches for patients with osteosarcoma. Major roadblocks include that PDXs often fail to engraft and require many months to obtain results, severely reducing clinical utility and substantially increasing expense (71–74). For example, in a trial conducted by Champions Oncology, 24% of sarcoma PDXs failed to engraft and an additional 21% of the patients succumbed to metastatic disease prior to PDX testing results became available (75). Similarly, in the highly immunodeficient NOD/SCID gamma mice (76), osteosarcoma PDX engraftment ranged from 36% to 50% (bioRxiv 2024.2007.2022.604648; refs. 6, 77, 78). Predictive power is further impaired because the PDXs often do not reflect heterogeneity within the tumors (79), are frequently implanted in non-orthotopic sites (80, 81), and because the extended time frame can alter drug responses by allowing genetic/epigenetic changes or infiltration of murine cells (71, 79, 82). In contrast, every patient sample that we tested successfully formed sarcospheres and screening results can be obtained within 3 weeks, a clinically actionable time frame.

Romidepsin substantially outperformed the other HDIs in our studies both with and without MAP chemotherapeutics. The superior effects of romidepsin compared with other HDIs are likely due to a combination of its unique mechanisms of action and its unique mechanisms of resistance (83–88). Unique features of romidepsin compared with other HDIs include that it requires intracellular activation by reduction of the intramolecular disulfide bond (56). Reduction of the disulfide increases romidepsin hydrophilicity likely causing increased intracellular retention (56). Perhaps more importantly, romidepsin has slow/tight binding kinetics (51, 52) that can lead to long-lasting effects after drug removal (50, 54, 89). Romidepsin also has unique sets of HDAC targets, transcriptional co-regulators, and non-histone target proteins (48, 53), as well as unique non-transcriptional effects (50, 53, 54, 90, 91). Future studies will determine which of these unique mechanisms account for romidepsin outperforming the other HDIs in our studies.

Our results are also consistent with previous findings that the primary response to nanomolar levels of romidepsin can be either cell cycle arrest or apoptosis depending on the cell line (54) and that romidepsin is more potent on cancer cell lines than on non-transformed cell lines (92, 93). Romidepsin can also inhibit growth of tumors and metastases during the period of drug administration in murine osteosarcoma models. For example, growth of LM7 cell–derived lung metastases was prevented when romidepsin administration continued for the entire 21-week observation period (94). In the same study, romidepsin had less effect on lung metastases derived from murine K7M2 osteosarcoma cells in syngeneic mice (94). In subcutaneous xenograft models of osteosarcoma cell lines, tumors regressed when mice were followed for 1 week after discontinuation of romidepsin administration (95). Romidepsin also inhibited growth of well-established subcutaneous xenografts in a small number of mice but did not induce a sustained response when the mice were followed for 6 weeks after romidepsin discontinuation (96). Taken together, romidepsin seems effective in multiple murine osteosarcoma models during the period of administration but not after discontinuation. That finding is consistent with how romidepsin is used clinically as it is administered to patients with cutaneous T-cell lymphoma for as long as they continue to benefit. Nonetheless, a limitation of our study is the lack of experiments to assess *in vivo* effects of romidepsin in murine osteosarcoma models. Future studies will determine whether romidepsin inhibits growth of metastases derived from our low-passage patient-derived osteosarcoma cells in murine models.

A likely clinical application for romidepsin in osteosarcoma is combination with MAP (25, 97). Combination therapy can increase survival and decrease both the development of resistance and the required dosages of MAP (97). Romidepsin had additive-to-synergistic activity with MAP in our sarcosphere experiments but the lack of clinically relevant dosing of MAP is a limitation of this study. Other HDIs synergize with doxorubicin or cisplatin in osteosarcoma cells (61, 63, 98–101) and romidepsin does in other cancers (102, 103). Further studies should be done to determine whether romidepsin synergizes individually with either methotrexate, doxorubicin, or cisplatin using clinically relevant dosing schemes to determine whether romidepsin could potentially reduce the amount of one of the MAP chemotherapeutics administered to patients and thereby reduce lifelong toxicity (104, 105). Romidepsin causes few severe toxicities (106). Nausea is the most common side effect and other gastrointestinal side effects such as vomiting and anorexia can occur. However, these side effects were not severe in most patients (106). The most common severe adverse events are thrombocytopenia and neutropenia (106). The most concerning potential side effect of romidepsin is the cardiac toxicity that has been reported in humans, rats, and beagle dogs (49, 106, 107). However, grade 3 or 4 cardiac toxicities are rare when patients with cardiac risk factors are excluded and serum levels of potassium and magnesium are normalized prior to each administration of romidepsin (106–108). Because of the adverse cardiac toxicities associated with doxorubicin (104), this is a potential limitation for combining romidepsin with doxorubicin. Future *in vivo* studies and clinical trials would be needed to determine whether overlapping toxicities will necessitate dose modifications were romidepsin combined with one or more of the MAP chemotherapeutics.

Together, our sarcosphere studies support further investigation of romidepsin as a potential therapeutic for osteosarcoma lung metastases. Romidepsin had higher Cmax/ED_50_ ratios and higher therapeutic indices (TD_50_/ED_50_ ratios) than all other drugs in the NCI panel of FDA-approved drugs, the three other FDA-approved HDIs, and another eight HDIs that are in clinical trials. Clinically tolerable levels of romidepsin also substantially reduced metabolic activity in sarcospheres derived from cells of ∼30% of human and 50% of canine patients with osteosarcoma with ED_50_s less than 70 nmol/L. That selectivity suggests that sarcospheres may be useful to identify which patients with osteosarcoma are most likely to benefit from romidepsin. That approach should be validated in murine models and could inform canine and human trials. Canine trials would also act as preclinical studies prior to human trials as osteosarcoma is highly similar in the two species and the prevalence in canines is substantially higher than in humans (25, 27, 28).

## Supplementary Material

Figure S1 Dose responses and ED50s from secondary screen

Figure S2 Dose responses and Bliss Prediction of Additivity for LM7 sarcospheres in secondary screen

Figure S3 Dose responses and Bliss Prediction of Additivity for 143B sarcospheres in secondary screen

Figure S4 Dose Responses and Bliss Prediction of Additivity for MG63.3-GFP sarcospheres in secondary screen

Figure S5 Toxicity and therapeutic indices on Normal Human Osteoblasts

Figure S6 Toxicity and therapeutic indices on Small Airway Epithelial Cells

Figure S7 Screen of HDIs in clinical trials

Figure S8 Dose responses for HDIs on 143B, LM7, and MG63.3-GFP sarcospheres

Figure S9 The patient-derived cells have numerous genomic amplifications and deletions, consistent with derivation of the cells from the osteosarcoma itself, rather than from bystander cells

Figure S10 Sarcospheres derived from cells of a canine patient do not grow over time and romidepsin does not affect diameter

Table S1 Suppliers and Storage Conditions for 13 Drugs in Secondary Screen

Table S2 Concentrations of MAP Chemotherapeutics Used (nM)

Table S3 Cmax and Sarcosphere ED50s for 13 Hits in Secondary Screen and Histone Deacetylase Inhibitors

## Data Availability

Data were generated by the authors and are available upon request from the corresponding author.
