# Supplementary figures and images for: Screening FDA-Approved Oncology Drugs with Three-Dimensional Spheroids Identifies Romidepsin as a Therapeutic Candidate for Osteosarcoma
Source: Cancer Res Commun. 2025 Oct 15;5(10):1821–38. doi: 10.1158/2767-9764.CRC-25-0121 (PMC12522106; doi:10.1158/2767-9764.CRC-25-0121)

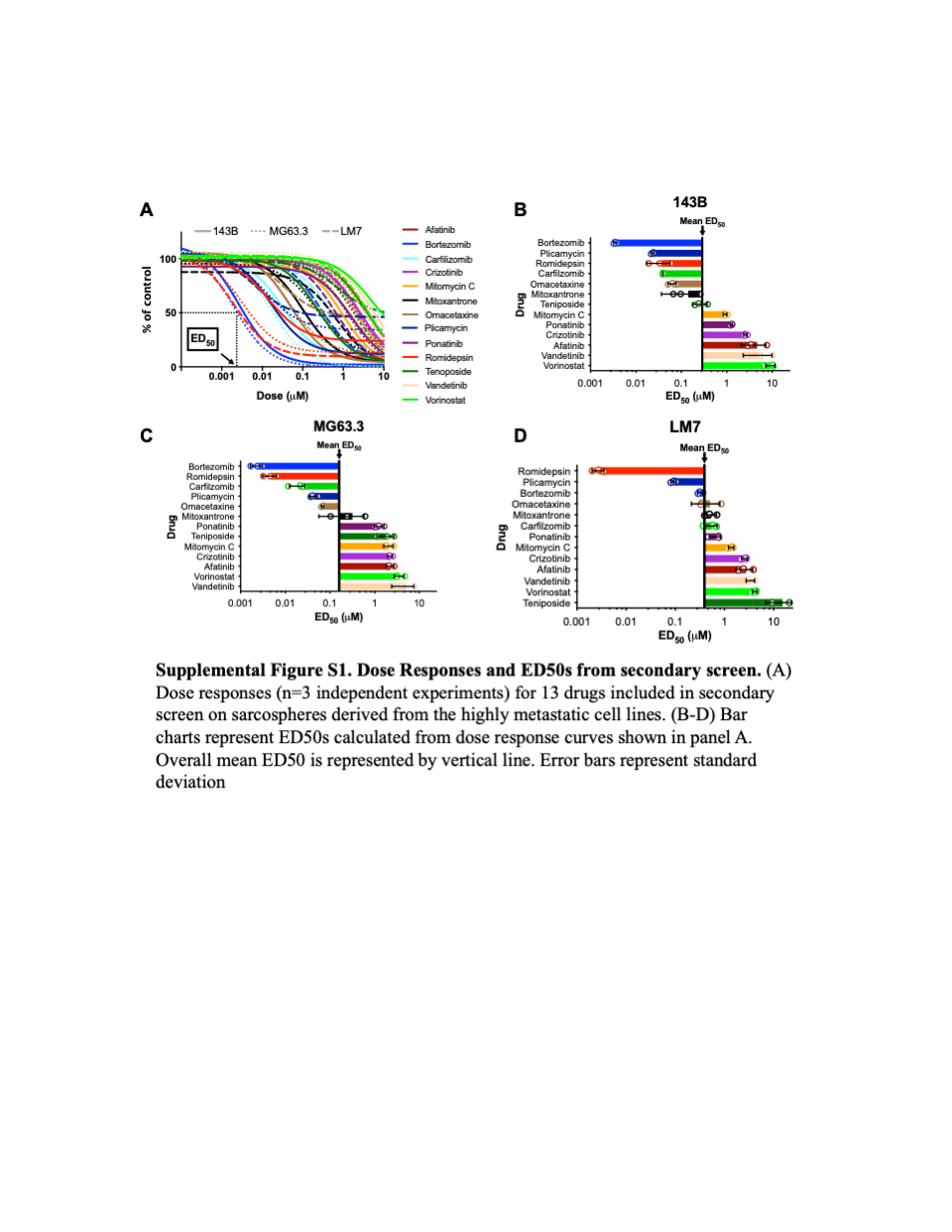

Supplement: Figure S1 — Dose responses and ED50s from secondary screen [file crc-25-0121_figure_s1_suppsf1.png]

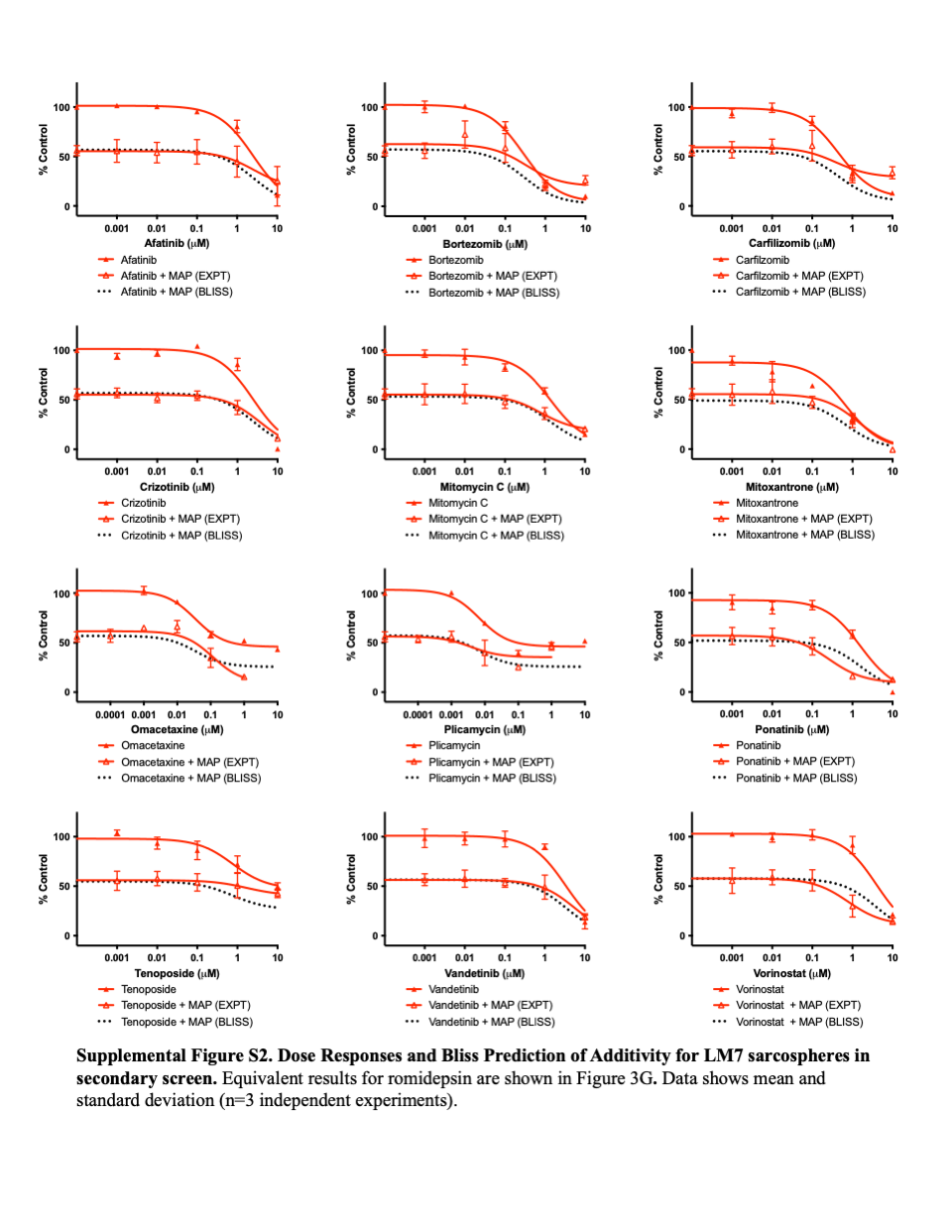

Supplement: Figure S2 — Dose responses and Bliss Prediction of Additivity for LM7 sarcospheres in secondary screen [file crc-25-0121_figure_s2_suppsf2.png]

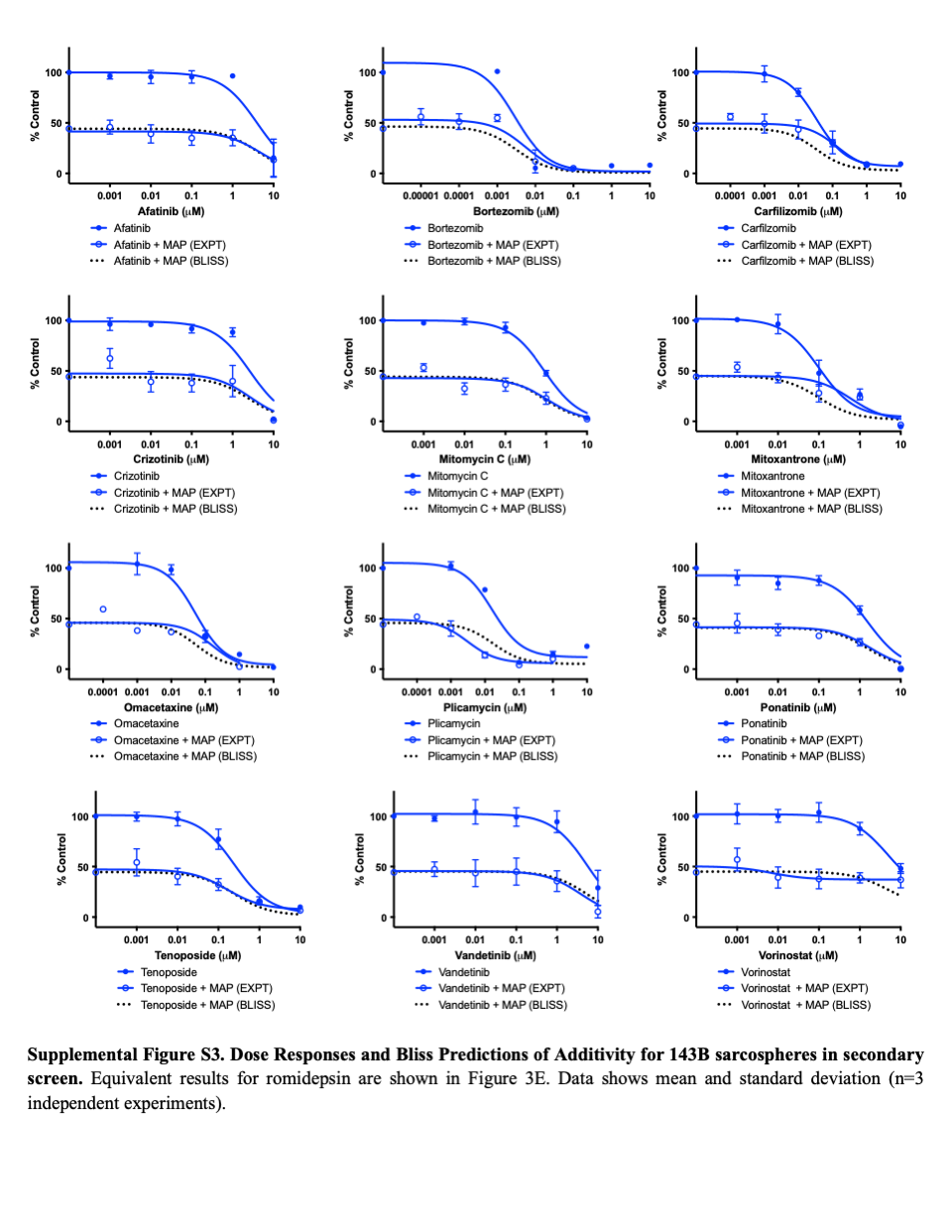

Supplement: Figure S3 — Dose responses and Bliss Prediction of Additivity for 143B sarcospheres in secondary screen [file crc-25-0121_figure_s3_suppsf3.png]

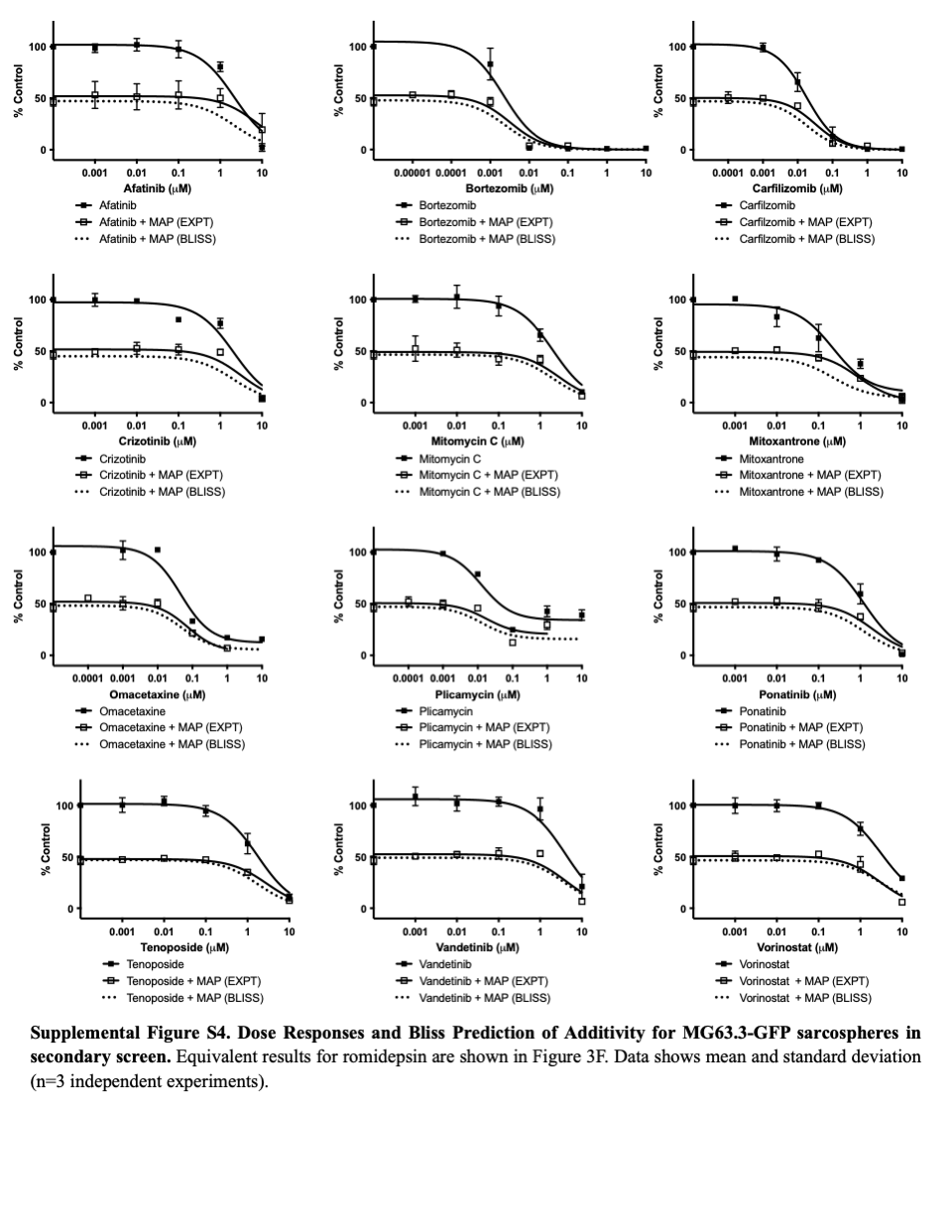

Supplement: Figure S4 — Dose Responses and Bliss Prediction of Additivity for MG63.3-GFP sarcospheres in secondary screen [file crc-25-0121_figure_s4_suppsf4.png]

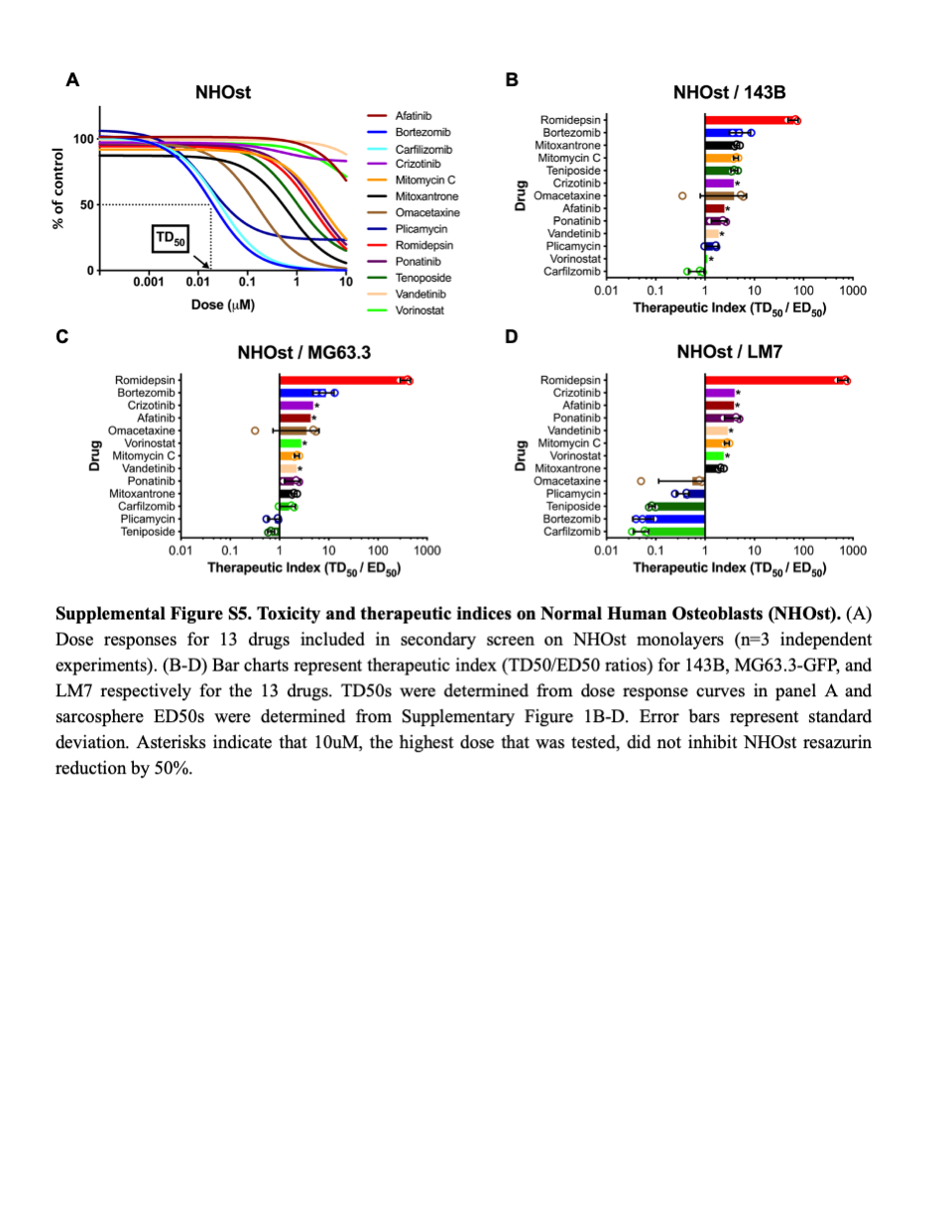

Supplement: Figure S5 — Toxicity and therapeutic indices on Normal Human Osteoblasts [file crc-25-0121_figure_s5_suppsf5.png]

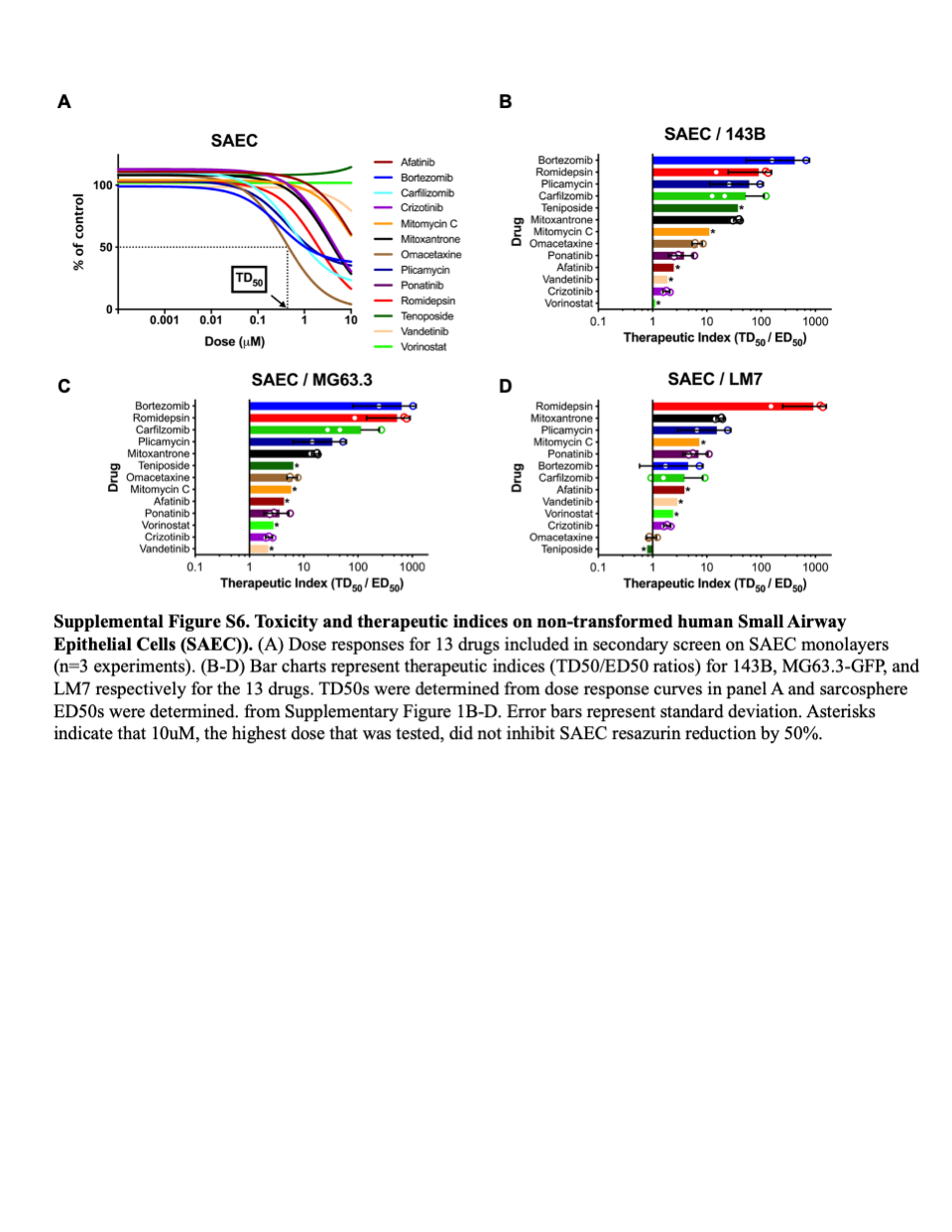

Supplement: Figure S6 — Toxicity and therapeutic indices on Small Airway Epithelial Cells [file crc-25-0121_figure_s6_suppsf6.png]

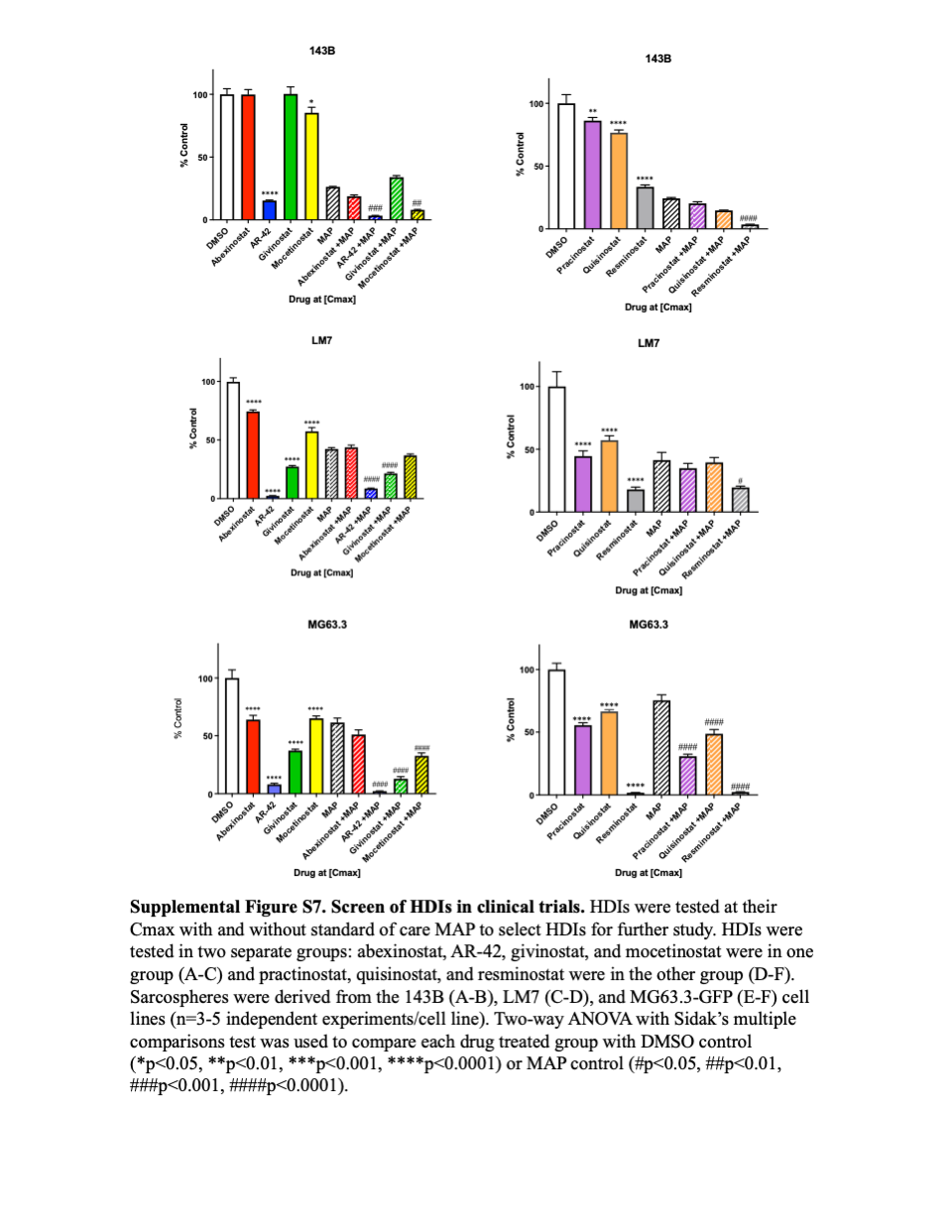

Supplement: Figure S7 — Screen of HDIs in clinical trials [file crc-25-0121_figure_s7_suppsf7.png]

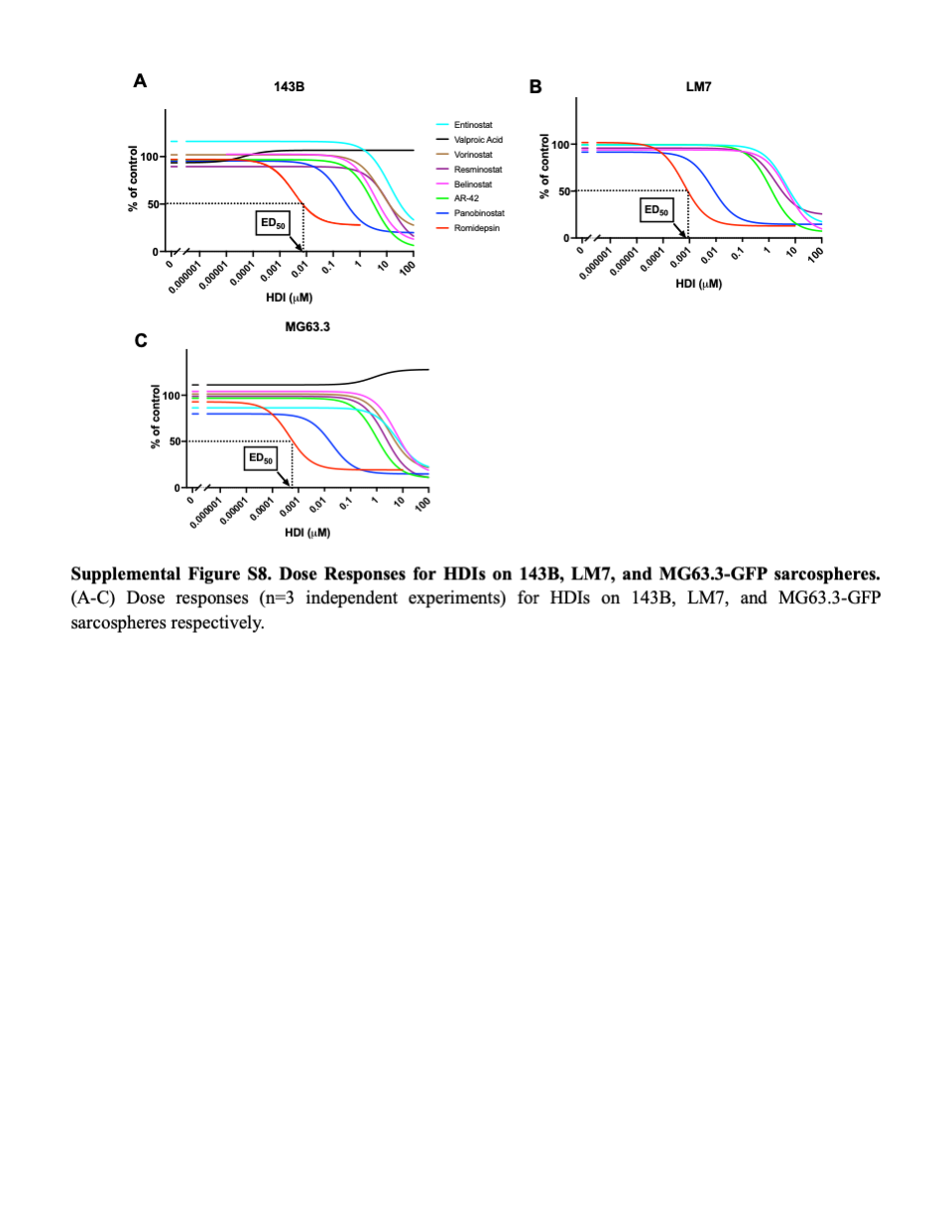

Supplement: Figure S8 — Dose responses for HDIs on 143B, LM7, and MG63.3-GFP sarcospheres [file crc-25-0121_figure_s8_suppsf8.png]

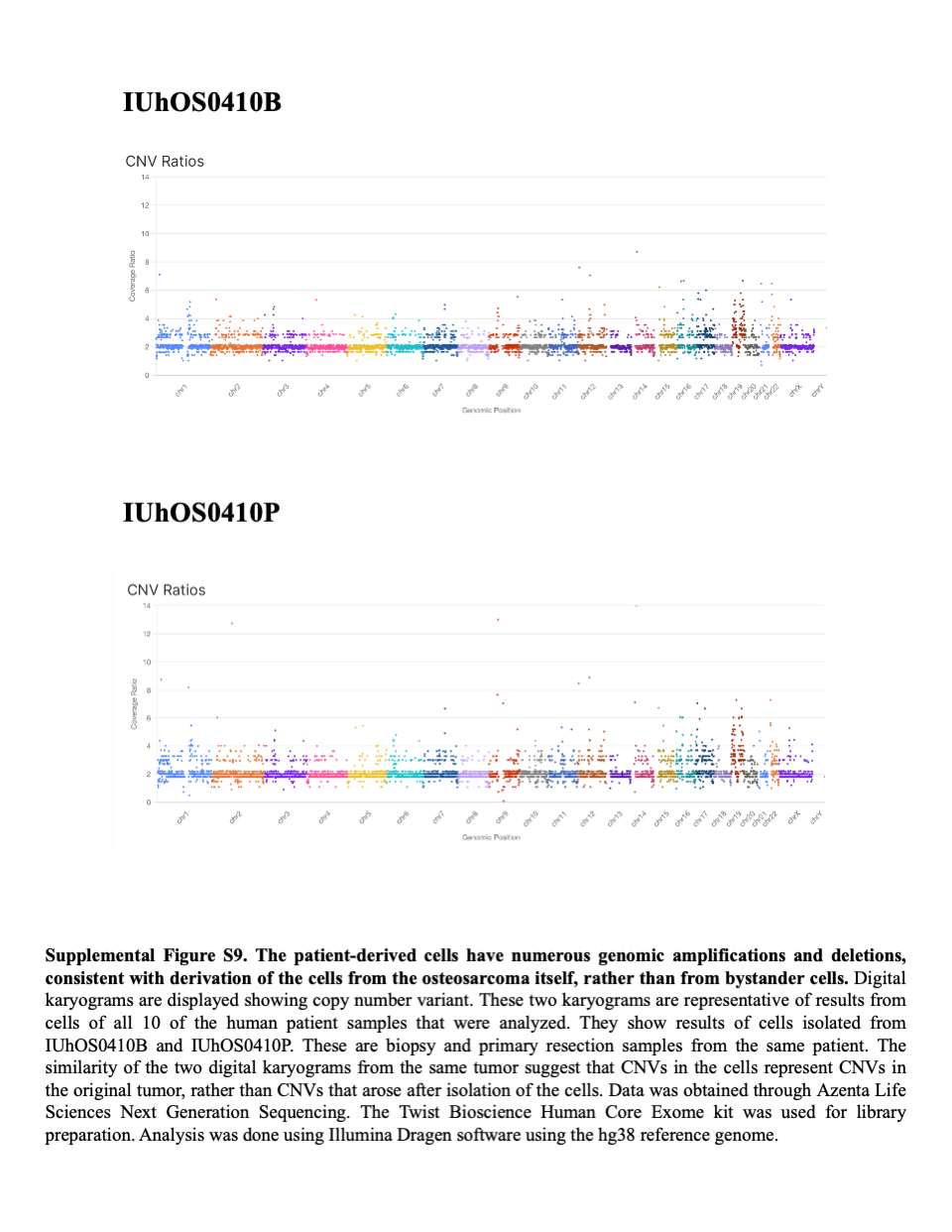

Supplement: Figure S9 — The patient-derived cells have numerous genomic amplifications and deletions, consistent with derivation of the cells from the osteosarcoma itself, rather than from bystander cells [file crc-25-0121_figure_s9_suppsf9.png]

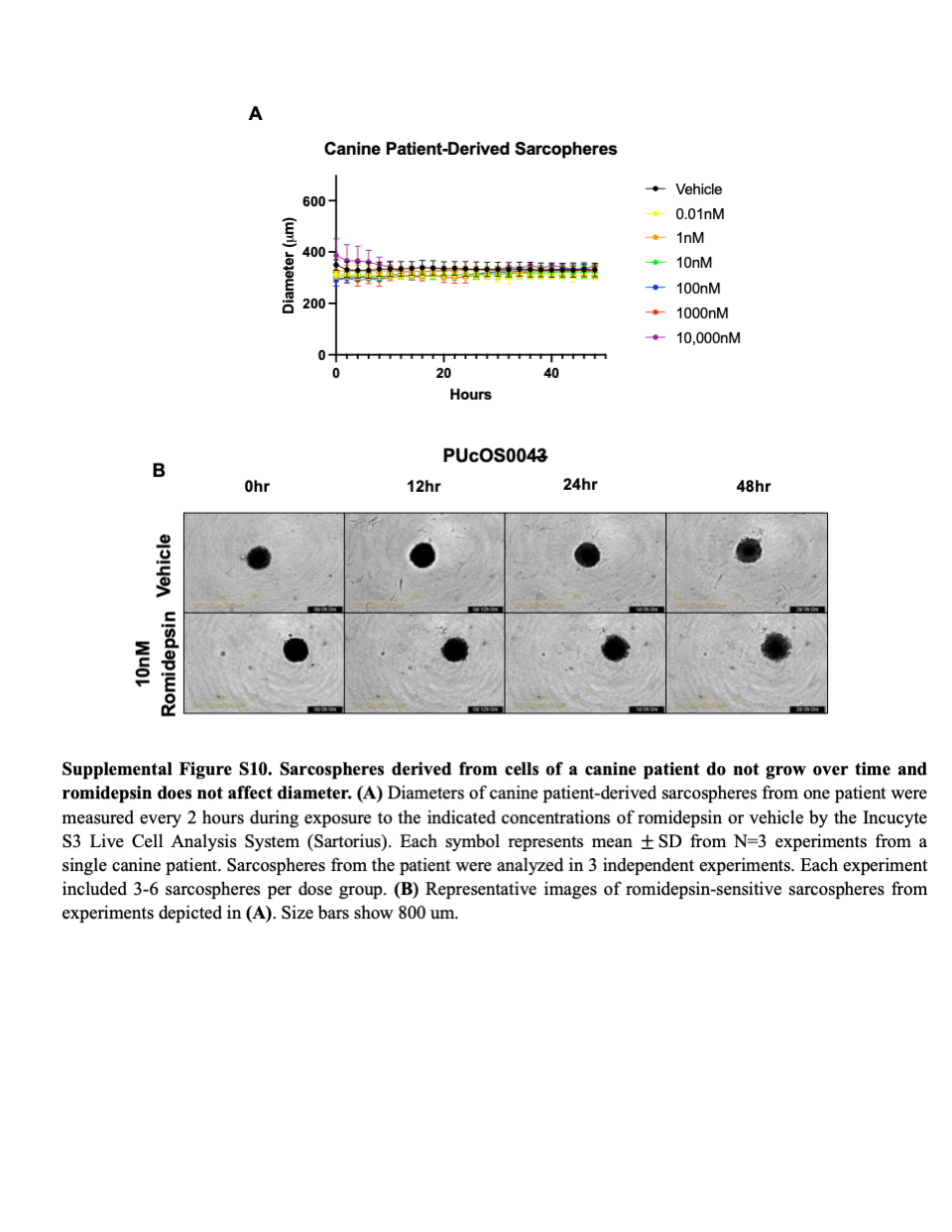

Supplement: Figure S10 — Sarcospheres derived from cells of a canine patient do not grow over time and romidepsin does not affect diameter [file crc-25-0121_figure_s10_suppsf10.png]
