# Supplementary material for: Screening FDA-Approved Oncology Drugs with Three-Dimensional Spheroids Identifies Romidepsin as a Therapeutic Candidate for Osteosarcoma
Source: Cancer Res Commun. 2025 Oct 15;5(10):1821–38. doi: 10.1158/2767-9764.CRC-25-0121 (PMC12522106; doi:10.1158/2767-9764.CRC-25-0121)
Supplement: Supplementary file 2 [file crc-25-0121_table_s1_suppst1.docx]

Supplemental Table 1. Suppliers and Storage Conditions for 13 Drugs in Secondary Scre

Drug	Supplier	Sto
Bortezomib / PS-341	Boston Biochem / R&D	-2
Romidepsin / FK-228	Tocris / R&D	-2
Plicamycin / NSC-24559	DTP / Sigma	Dark
Mitomycin C / NSC-26980	DTP/ APAC Pharm	Darl
Teniposide / NSC-122819	DTP / Sigma	Dark
Mitoxantrone / NSC-279836	DTP	-2
Vorinostat / NSC-701852	DTP / Sigma	-2
Afatinib / NSC-750691	DTP	-2
Crizotinib / NSC-756645	DTP	Desicca
Carfilzomib / NSC-758252	DTP	Desiccate,
Omacetaxine / NSC-758253	DTP	-2
