# Supplementary material for: Screening FDA-Approved Oncology Drugs with Three-Dimensional Spheroids Identifies Romidepsin as a Therapeutic Candidate for Osteosarcoma
Source: Cancer Res Commun. 2025 Oct 15;5(10):1821–38. doi: 10.1158/2767-9764.CRC-25-0121 (PMC12522106; doi:10.1158/2767-9764.CRC-25-0121)
Supplement: Supplementary file 3 [file crc-25-0121_table_s2_suppst2.docx]

**Supplemental Table 2. Concentrations of MAP Chemotherapeutics Used (nM)**

**Initial Screen: To obtain GIC20 with methotrexate, doxorubicin, and cisplatin in combination[illegible]**

| Drug | LM7 | 143B | MG63[illegible] |
|---|---|---|---|
| Methotrexate | 61,091 | 4.83 | 8,21[illegible] |
| Doxorubicin | 27.9 | 48.3 | 58 |
| Cisplatin | 104.7 | 108.7 | 154.[illegible] |

**Secondary Screen: To obtain ED50 with methotrexate, doxorubicin, and cisplatin in combinati[illegible]**

| Drug | LM7 | 143B | MG63[illegible] |
|---|---|---|---|
| Methotrexate | [illegible] | [illegible] | [illegible] |
